# Phylogenetic analysis of the human thyroglobulin regions

**DOI:** 10.1186/1756-6614-5-3

**Published:** 2012-05-01

**Authors:** Abdelaziz Belkadi, Caroline Jacques, Frédérique Savagner, Yves Malthièry

**Affiliations:** 1 INSERM U694, Institut Biologie Santé (IBS), rue des Capucins, F-49100 Angers, France; 2 University of Angers, rue de Rennes, F-49045 Angers, France; 3 Laboratory of human genetics of infectious diseases. Necker Branch, INSERM, U980, Paris, France

## Abstract

Thyroglobulin is a large protein present in all vertebrates. It is synthesized in the thyrocytes and exported to lumen of the thyroid follicle, where its tyrosine residues are iodinated . The iodinated thyroglobulin is reintegrated into the cell and processed (cleaved to free its two extremities) for thyroid hormone synthesis. Thyroglobulin sequence analysis has identified four regions of the molecule: Tg1, Tg2, Tg3 and ChEL. Structural abnormalities and mutations result in different pathological consequences, depending on the thyroglobulin region affected. We carried out a bioinformatic analysis of thyroglobulin, determining the origin and the function of each region. Our results suggest that the Tg1 region acts as a binding protein on the apical membrane, the Tg2 region is involved in protein adhesion and the Tg3 region is involved in determining the three-dimensional structure of the protein. The ChEL domain is involved in thyroglobulin transport, dimerization and adhesion. The presence of repetitive domains in the Tg1, Tg2 and Tg3 regions suggests that these domains may have arisen through duplication.

## Introduction

Thyroglobulin is the precursor of the thyroid hormones triiodothyronine (T3) and thyroxine (T4). In humans, thyroglobulin is synthesized by thyroid follicle cells, which are also known as thyrocytes [1]. Thyroglobulin molecules form dimers, which are exported to the lumen of the thyroid follicles [2]. There, the thyroglobulin is immobilized on the apical membrane. The thyroid hormones process starts by the iodination of tyrosine residues. Thyroperoxidaseis activated by *H*_2_*O*_2_, leading to the oxidation of iodide, followed by the iodination and conjugation of some of the tyrosine residues present in the thyroglobulin molecule. The iodinated and conjugated thyroglobulin is then returned to the cell via an endocytosis process that may involve histone H1 [3], megalin (gp330) [4] and/or the N-acetylglucosamine receptor [5]. Only a very small number of iodinated tyrosine residues are involved in thyroid hormone synthesis. T4 is formed by the conjugation of two residues of diiodotyrosine followed by cleavage. T3 is formed in a similar manner, but through the conjugation of diiodotyrosine with monoiodotyrosine [6,7]. T3 is the functional form; it is generated principally by T4 deiodinases in the peripheral organs, with only 13% being formed in the thyroid gland [8]. Thyroid hormones reach their target organs via the bloodstream. Thyroglobulin has been reported to regulate some thyroid genes and the growth of epithelial cells [9,10]. It acts as both a hormone and an iodine reservoir [11].

**Figure 1:**
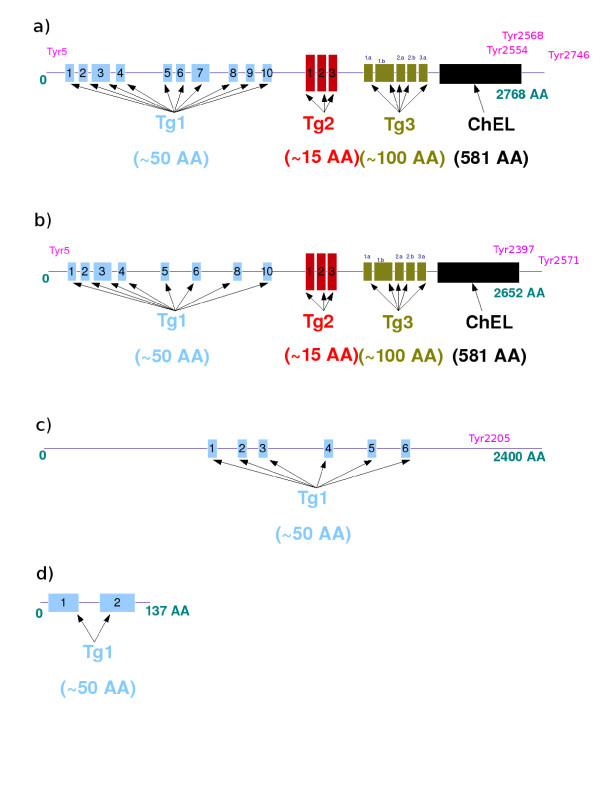
**The structure of the thyroglobulin protein.a)** Structure of the human thyroglobulin protein. **b)** Structure of the zebrafish thyroglobulin protein. **c)** Structure of the amphioxus thyroglobulin-like protein. **d)** Structure of the sea urchin thyroglobulin-like protein. Blue: Tg1 domains, red: Tg2 domains, green: Tg3 domains, black: the ChEL domain. Magenta: the location of the thyroid hormone synthesis sites on the proteins (tyrosine residues).

In humans, mice and fish, thyroid hormone levels determine the basal rate of metabolism and overall energy expenditure [12-14]. In other species, such as Senegalese sole [15], amphibians [16], urochordatas [17], amphioxus [18] and lamprey [19], thyroid hormones play a critical role in the metamorphosis from larvae to juveniles. Thyroglobulin protein structure has been studied in detail [20-22]. This protein is present in all vertebrates and always has the same structure, consisting of four regions: the Tg1 (∼ 10 repetitive domains), Tg2 (3 repetitive domains), Tg3 (5 repetitive domains) and ChEL regions (Figure 1-a and 1-b). The Tg1, Tg2 and Tg3 regions (moving along the molecule from its N-terminal end) consist of repetitive domains. All three regions are rich in cysteine residues, allowing them to form disulfide bonds [23]. The presence of these repetitive domains suggests their possible evolution through the duplication of source domains. The C-terminus of the molecule includes a 581-amino acid sequence displaying a high degree of similarity to the sequence of acetylcholinesterase (28% identity) [24,25]. One previous study identified the ChEL domain as the origininal source of thyroglobulin [26]. Thyroglobulin contains about 140 tyrosine residues, but only about 30 of these residues are iodinated and a very small number of these iodinated tyrosines undergo conjugation to form T3 and T4 [27]. Only four major thyroid hormone synthesis sites have been clearly identified in the human thyroglobulin molecule and these sites are located at either end of the protein: Tyr5, Tyr2554, Tyr2568 and Tyr2747 [21].

Thyroglobulin may thus be seen as a huge precursor of two very small products. Additional studies of its other, as yet unexplored functions in the cell may be useful. For example, this protein could potentially be involved in the trafficking of iodinefrom the thyrocyte to the follicle lumen and its storage. Many studies have made use of bioinformatics tools to analyze the evolution of proteins and genes, and such tools may be useful in this context [28,29].

We performed a phylogenetic analysis of the thyroglobulin molecule with the sequenced genomes of species corresponding to key steps in animal evolution. Our results provide clues to the evolution of thyroglobulin and potential functional roles for theTg1, Tg2, Tg3 and the ChEL regions.

## Materials and methods

### Sequence extraction

We extracted the available DNA and protein sequences for thyroglobulin (Tg) from the NCBI databank http://www.ncbi.nlm.nih.gov for four species: human [GenBank:CAA29104],rat [GenBank:AAF34909], mouse [GenBank:AAB53204], pig [GenBank:ACY66900]. We also extracted six predicted sequences: cattle [GenBank:NP_ 776308] horse [GenBank:XP 001916622] marmoset [GenBank:XP 002759270], panda [Gen-Bank:XP 002917659], zebrafish [GenBank:XP 694292] and zebra finch [GenBank:XP 002188056]. For other species, for which the amino-acid sequence of is unknown, such as opossum, and fugu, we used the human thyroglobulin genomic sequence in Blast searches of the UCSC website genome.ucsc.edu; We first translated the DNA sequence to obtain a putative amino-acid sequence. We then used Blast to check whether the predicted sequence was present in the database (chr3:411,623,333-412,004,486 and chrUn:270,007,531-270,025,053 for opossum and fugu, respectively). Homologous sequences from amphioxus [GenBank:XP 002607132] and sea urchin [GenBank:XP 001202473] were also identified by BLAT analysis (chrUn:353,044,426-353,083,914 and Scaffold82420:233-1,088 in the amphioxus and sea urchin genomes, respectively).

## Sequence similarity

We searched for regions presenting sequence similarities to the constituent domains of thyroglobulin - Tg1 Tg2, Tg3 and ChEL - with the Blastall command ftp://ftp.ncbi.nlm.nih.gov/blast/db/, version 2.2.19. Pairs of sequences were compared on the basis of their global alignment with the Myers & Millers algorithm manpages.ubuntu.com/manpages/karmic/man1. Results were generated in a separate text file containing alignment diagrams, scores, degrees of identity, similarity and gaps. We used ClustalX software ftp://ftpigbmc.u-strasbg.fr/pub/ClustalX/. for analysis of multiple alignments of three or more sequences. The results were output to a separate text file, but without information about score, because it was not possible to use more than two sequences for score calculation with Compositional Matrix Adjust.

### Phylogeny

We used the neighbor-joining (NJ) method in PHYLIP [30] and mega 5 [31] for phylogenetic analysis. A range of analyses, from simple p distance to multiparameter models with gamma correction, were used. The significance of the phylogenetic tree was assessed by bootstrapping, with 10,000 iterations. The Jones-Taylor- Thornton (JTT) model of amino-acid sequence evolution, with gamma correction, was used for distance estimation [32]. In each case, the distance was validated with 10,000 bootstrap replications.

## Results

### The N-terminal Tg1 region

**Figure 2:**
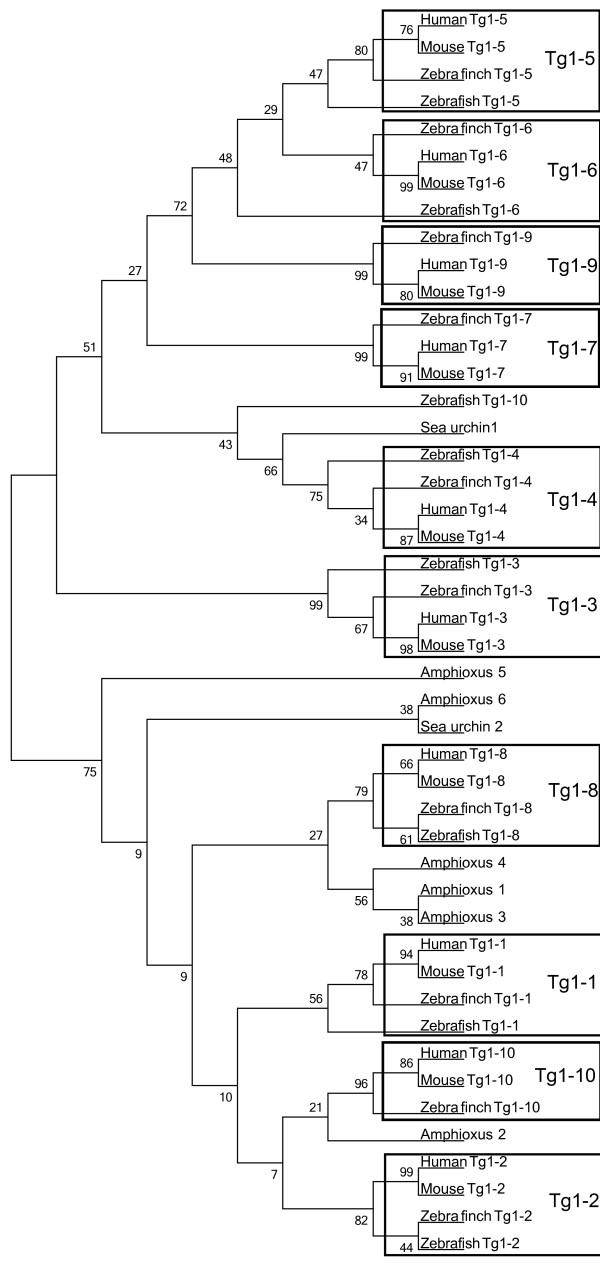
**Phylogenetic analysis of thyroid hormone precursor Tg1 domains for six species.** Phylogenetic analysis of thyroglobulin Tg1 domains from 4 species (human, mouse, zebra finch and zebrafish) and the amphioxus and sea urhin thyroglobulin-like Tg1 domains. Evolutionary history was inferred by the neighbor-joining method. The bootstrap consensus tree inferred from 1000 replicates is taken to represent the evolutionary history of the taxa analyzed. The numbers at nodes representing bootstrap support scores.

**Figure 3:**
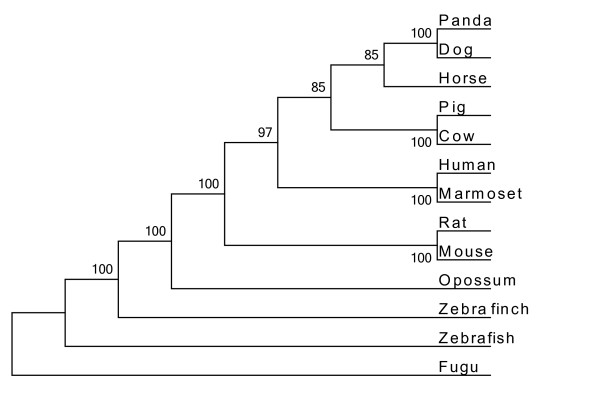
**Phylogenetic analysis of thyroglobulin Tg1 regions from 13 species.** Phylogenetic analysis of thyroglobulin Tg1 regions from 13 species (human, marmoset, rat, mouse, panda, dog, horse, pig, cow, opossum, zebra finch, zebrafish and fugu). Evolutionary history was inferred by the neighbor-joining method. The bootstrap consensus tree inferred from 1000 replicates is taken to represent the evolutionary history of the taxa analyzed. The numbers at nodes representing bootstrap support scores.

**Figure 4:**
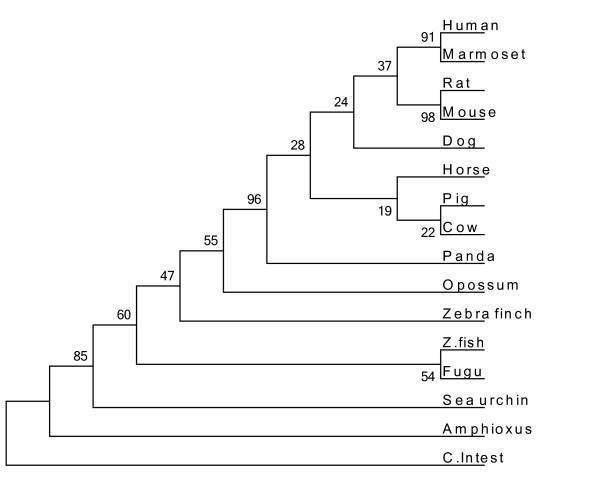
**The phylogenetic analysis of thyroglobulins from 13 species.** Phylogenetic analysis of thyroglobulins from 13 species (human, marmoset, rat, mouse, panda, dog, horse, pig, cow, opossum, zebra finch, zebrafish and fugu) and of thyroglobulin-like proteins from Ciona intestinalis, amphioxus and sea urchin. Evolutionary history was inferred by the neighbor-joining method. The bootstrap consensus tree inferred from 1000 replicates is taken to represent the evolutionary history of the taxa analyzed. The numbers at nodes representing bootstrap support scores.

In humans, the first region of thyroglobulin consists of 10 Tg1 repeat domains, each containing 50 amino acids and displaying 14% identity. However, Molina, et al. identified an 11^th^ domain located after the Tg2 region [33]. A comparison of this region in all the thyroglobulin protein sequences extracted (13 species) indicated that the fish thyroglobulins (zebrafish and fugu) lacked Tg1-7 and Tg1-9 (Additional file 1: Figure S1). We used mega 5 software to calculate the distance of the whole thyroglobulin protein sequences and of each of the component regions (Additional file 2: Tables S2, Additional file 3: Table S3, Additional file 4: Table S4 and Additional file 5:Table S5). We performed a phylogenetic analysis on the thyroglobulin Tg1 domains of four vertebrate species - (human (10 Tg1 domains), mouse (10 Tg1 domains), zebra finch (10 Tg1 domains) and zebrafish (8 Tg1 domains)) - six Tg1 domains from amphioxus (a cephalochordate) and two Tg1 domains from sea urchin (an echinoderm) (Figure 2). The sixth amphioxus Tg1 domain clusteredwith the second sea urchin domain in the phylogenetic tree. With a lower bootstrap percentage, we observed two big major branches of the phylogenetic tree, the first corresponding to the sea urchin and amphioxus Tg1 domains, which clustered with the thyroglobulin Tg1-8, Tg1-2, Tg1-1 and Tg1-10 domains, and the second corresponding to the Tg1-3, Tg1-4, Tg1-7, Tg1-5, Tg1-9 and Tg1-6 domains. For confirmation of these results, we performed a phylogenetic analysis on the thyroglobulin Tg1 domains of 13 vertebrate species (human (10 Tg1 domains), marmoset (10 Tg1 domains), pig (10 Tg1 domains), horse (10 Tg1 domains), dog (10 Tg1 domains), panda (10 Tg1 domains), rat (10 Tg1 domains), mouse (10 Tg1 domains), cow (10 Tg1 domains), opossum (10 Tg1 domains), zebra finch (10 Tg1 domains), zebrafish (8 Tg1 domains) and fugu (8 Tg1 domains)) together with six Tg1 domains from amphioxus and two from sea urchin. This new tree also had two major branches (Additional file 6: Figure S2). The fish Tg1-10 domains did not cluster with the other Tg1-10 domains in either of the trees. We also investigated the genome of the urochordate Ciona intestinalis. The protein with the largest number of Tg1 motifs was a predicted protein (rather than one for which the amino-acid sequence was actually known similar to entractin/nidogen (XP_ 002125504.1) and containing three Tg1 motifs. We generated two phylogenetic trees, one based on 13 vertebrate Tg1 regions (human, marmoset, pig, horse, dog, panda, rat, mouse, cow, opossum, zebra finch, zebrafish and fugu) (Figure 3) and the second based on 13 vertebrate thyroglobulin proteins (human, marmoset, pig, horse, dog, panda, rat, mouse, cow, opossum, zebra finch, zebrafish and fugu) together with the sequences of Thyroglobulin homologs from Ciona intestinalis, amphioxus and sea urchin (Figure 4).

We investigated the function of the Tg1 region of thyroglobulin, by investigating proteins containing domains similar to the Tg1 domain with cutoff e-value = 0.15 as recommended by the software. For the Tg1-1 domain, 107 proteins were selected (15 thyroglobulins, 15 nidogens, 18 testicans, 16 secreted proteins, acidic, cysteine-rich (SPARC) proteins, 13 invariant chains and 30 unnamed or hypothetical proteins). For the Tg1-2 domain, 49 proteins were retained (15 thyroglobulins, 13 nidogens, 3 testicans, 2SPARC proteins, 3 invariant chains and 13 unnamed or hypothetical proteins). For the Tg1-3 domain, 16 proteins were found (thyroglobulins only). For the Tg1-4 domain, we retained 97 proteins (16 thyroglobulins, 13 nidogens, 10 testicans, 13 SPARC proteins, 4 invariant chains, 1 insulin-like growth factor binding protein (IGFBP) and 40 unnamed or hypothetical proteins). For the Tg1-5 domain, 103 proteins were retained (18 thyroglobulins, 6 nidogens, 11 testicans, 19 SPARC proteins, 3 invariant chains,2 IGFBPs and 44 unnamed or hypothetical proteins). For the Tg1-6 domains, 49 proteins were identified (17 thyroglobulins, 14 nidogens, 3 invariant chains and 15 unnamed or hypothetical proteins). For the Tg1-7 domain, 30 proteins were retained (10 thyroglobulins, 11 nidogens and 9 unnamed or hypothetical proteins). For the Tg1-8 domain, 105 proteins were retained (17 thyroglobulins, 13 nidogens, 9 testicans, 17 SPARC proteins, 5 invariant chains and 44 unnamed or hypothetical proteins). For the Tg1-9 domain, 11 proteins were found (thyroglobulins only). For the Tg1-10 domain, 100 proteins were retained (17 thyroglobulins, 13 nidogens, 7 testicans, 15 SPARC proteins, 3 invariant chains and 45 unnamed or hypothetical proteins). The number of thyroglobulin proteins displaying sequence similarity to the human Tg1 domains varied from 15 to 17, essentially due to the presence of incomplete thyroglobulin protein sequences in the databases we used, particularly for bears. The abovementioned proteinsdisplayed sequence similarities to the Tg1 regions of proteins from five families [34]: testicans, SPARC-related modular calcium binding (SMOC) proteins, nidogens, IGFBPs and invariant chains. Testican proteins are involved in the regulation of cell attachment, cysteine protease and metalloprotease activities [35-38]. SMOC proteins are glycoproteins present principally at the basement membrane and involved in the regulation of calcium binding [39,40]. SMOC and testican proteins are present in metazoans. Proteins of the nidogen family are known to control the three-dimensional structure of the basal membrane [41]. Nidogen proteins arealso involved in cell attachment, neutrophil chemotaxis and nervous system development [42,43]. IGFBP belongs to a family of seven proteins with high affinity for IGF with different functions in several tissues [44]. Nidogen and IGFBP are present in both tunicates and craniates. The invariant chain is involved in MHC-II cell formation [45]. This protein, like the thyroglobulin protein, is present only in vertebrates.

### The Tg2 region

The second region consists of three Tg2 repetitive domains of 15 amino acids each, presenting 24% identity. The phylogenetic analysis of this region was less robust than that of the Tg1 region, due to the small size of these domains. However, we identified 33 proteins displaying sequence similarity to the Tg2 region. Nine were thyroglobulins: Bos taurus, Mus musculus, Rattus norvegicus, Macaca mulatta, Canis lupus familiaris, Equus caballus, Sus scrofa, Taeniopygia guttata and Danio rerio. Eleven were signal peptide - CUB domain - EGF-like (SCUBE) proteins (SCUBE3: Canis familiaris, Mus musculus, Homo sapiens, Macaca mulatta, Sus scrofa and Danio rerio and/or SCUBE1: Homo sapiens, Canis familiaris, Rattus norvegicus, Bos taurus and Danio rerio). The other 13 proteins were unnamed or hypothetical proteins. SCUBE proteins are known to involved in adhesion. Queries of the PFAM databas http://pfam.sanger.ac.uk identified a GCC2-GCC3 domain conserved in the Tg2 region of mouse and human thyroglobulins. The GCC2-GCC3 domain is also present in the human SVEP1 and mouse SCUB2 proteins.

### The Tg3 region

**Figure 5:**
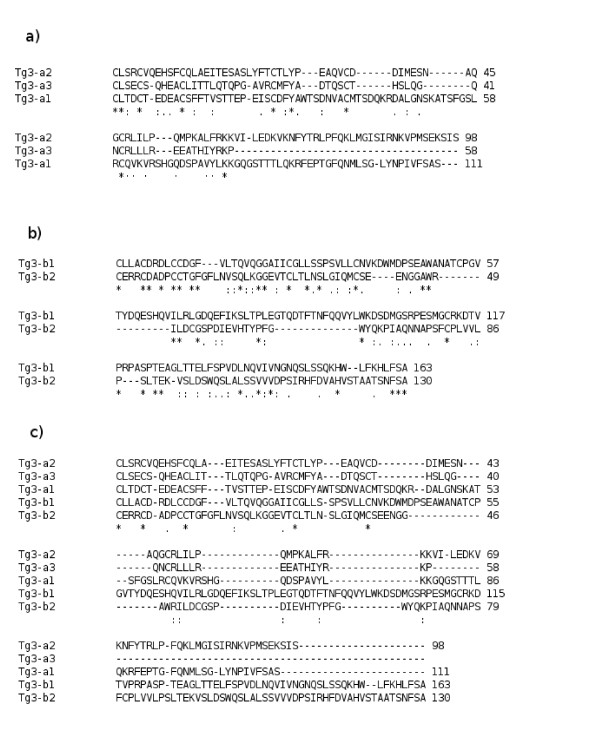
**Alignment of the Tg3 domains in human thyroglobulin.a)** The alignment of Tg3-a domains in human thyroglobulin: 83% of the cysteine residues are conserved in Tg3-a1, Tg3-a2 and Tg3-a3; 100% of the cysteine residues are conserved between Tg3-a1 and Tg3-a2. 55% of the conserved amino acids in Tg3-a domains are cysteine residues. **b)** The alignment of Tg3-b domains in human thyroglobulin: 100% of the cysteine residues are conserved in Tg3-b1 and Tg3-b2; 23% of the conserved amino acids in Tg3-b domains are cysteine residues. **c)** The alignment of all Tg3 domains in human thyroglobulin: 44% of the cysteine residues are conserved in all Tg3 domains; 100% of the conserved amino acids in Tg3 domains are cysteine residues.

In humans, the Tg3 region consists of five repetitive domains that can be classified into two subgroups: three domains in subgroup a (Tg3-a1: 111 AA, Tg3-a2: 98 AA, Tg3-a3: 58 AA) and two domains in subgroup b (Tg3-b1: 163 AA, Tg3-b2: 130 AA). TheseTg3 domains are 9% identical (Figure 5-a and 5-b). A search for proteins displaying sequence similarity to Tg3 domains identified only thyroglobulin proteins. Interestingly, the best conservation of cysteine residues between domains was observed in humans, with perfect conservation (100%) for Tg3-a domains and very high levels of conservation (87%) for Tg3-b domains. Furthermore, the five amino acids perfectly conserved in all Tg3 domains were cysteine residues (Figure 5-c). Cysteine residues account for 6% of all the amino acids present in the human Tg3 region and these residues were remarkably conserved in the thyroglobulin Tg3 regions of all the species studied; 100% of the 34 cysteine residues were perfectly conserved between the Tg3 regions of 12 species (human, rat, panda, marmoset, mouse, horse, dog, cow, zebra finch, zebrafish and fugu). In the opossum, two of the 34 cysteine residues in the Tg3 region were displaced (Additional file 1: Figure S1).

Tg3 domains were found only in vertebrate thyroglobulins. We investigated the origin of the Tg3 region domains, by comparing the sequences of the zebrafish Tg3 domains with the amphioxus protein. We found a similar sequence in region 413–441 of the amphioxus protein. Phylogenetic analysis including this region with the human and zebrafish thyroglobulin Tg3 domains clustered the 413–441 region of the amphioxus protein with the Tg3-2b domain of the human and the zebrafish thyroglobulins, albeit witha low bootstrap percentage (data not shown).

### The C-terminal ChEL domain

The ChEL domain of human thyroglobulin consists of 581 amino acids. This region displays a high level of similarity to acetylcholinesterase, hence its name. Acetylcholinesterase catalyzes the degradation of acetylcholine in the regulation of neurotransmission [46]. Blastall analyses of the ChEL domain identified 992 proteins displaying sequence similarity to this domain: 30 thyroglobulin proteins, 598 esterases (either carboxylesterases (n = 205) or cholinesterases (n = 150)) and 35 neuroligins. Cholinesterase-like regions have previously been identified in both enzymes and structural proteins [25]. When present in structural proteins, this region is thought to be related to cell movement, as a first sign of cell differentiation [47]. The function of the ChEL domain in thyroglobulin was recently linked to its transport throughout the endoplasmic reticulum [48]. Furthermore, ChEL-truncated thyroglobulin has been shown to be unable to form homodimers [49].

### Thyroid hormone synthesis sites

We determined the number of thyroid hormone synthesis sites in the thyroglobulin proteins studied here. The human thyroglobulin protein contains four major thyroid hormone synthesis sites [21]; An alignment of thyroglobulin sequences showed that the zebra finch, zebrafish and fugu proteins contained only three of the human thyroid hormone synthesis sites (Additional file 1: Figure S1). The first site (Tyr5) is the main site of hormone synthesis (more than 50%) [50] and was found to be present in all the thyroglobulin proteins studied. In amphioxus, the tyrosine residue in this position was replaced by a phenylalanine residue. Sequence alignment data showed that only the third site was present in amphioxus and that the Ciona intestinalis protein contained no thyroid hormone synthesis sites.

## Discussion

### The Tg1 region of thyroglobulin may be involved in binding

In vertebrates, iodination of the tyrosine residues of thyroglobulin requires the protein to be present in the lumen of the thyroid follicle. The iodinated thyroglobulin is then returned to the cell via a process called pinocytosis, which involves histone H1 [3], megalin (gp330) [4] and/or the N-acetylglucosamine receptor [5]. Our study of the thyroglobulin Tg1 region showed this region to be structurally related to proteins with binding functions from five families. Novinec et al [34] also described another protein with sequence similarity to the Tg1 domain, trophinin. This membrane protein has been shown to mediate the adhesion of homophilic cells [51]. We think the Tg1 region may mediate the binding of thyroglobulin to the thyrocyte apical membrane. The region of the H1 histone binding to thyroglobulin remains unidentified, whereas two regions of the N-acetylglucosamine receptor have been reported to bind thyroglobulin: RHL-1 subunit (N1-A500) [5] and (S789-M1,172) [52]. These receptors bind to the N-terminal end (Tg1 region) of the thyroglobulin protein. By contrast, megalin has been shown to interact with the carboxy-terminal domain of thyroglobulin, at R2,489-E2,503 [53], although the authors of this study were themselves critical of this work [54]. They reported that the region of interaction was poorly conserved between human and rat thyroglobulins and their finding that a rabbit antibody raised against R2,489-E2,503 reduced heparin-binding to rat Tg by only 70% led them to conclude that other heparin-binding sites must be involved in binding. These data, including the similarity of the Tg1 region to the extracellular matrix proteins nidogen and testican, provide support for our hypothesis that the Tg1 region is involved in the attachment and endocytosis of thyroglobulin.

### Phylogeny of the Tg1 region

The function of thyroglobulin seems to depend strongly on the follicle structure of the thyroid. This follicular structure is observed only in vertebrates. Nonetheless, although it remains unclear whether a colloid is present in the endostyle of the invertebrates of the chordate group, such as cephalochordates and urochordates, the endostyle is widely considered to be homologous to the follicle of the vertebrate thyroid gland [55]. This is not consistent with the detection of a thyroglobulin protein in Eisenia fetida by Wilhelm [56]. In annelids, hormones are produced exclusively by the central nervous system. No sequence that could be unambiguously identified as corresponding to a thyroglobulin was found in the amphioxus genome [57], but a large protein (about 2,400 amino acids) with biochemical properties similar to those of thyroglobulin has been described in this organism [58]. Both T3 and T4 have also been described in this cephalochordate [59]. The 2,400-amino acid thyroglobulin-like protein of this species contains six domains displaying sequence similarity to the Tg1 region but not to the Tg2, Tg3 and ChEL domains (Figure 1-c).

Another smaller protein of about 137 amino acids that clusters with vertebrate thyroglobulin in phylogenetic analysis was identified in sea urchin (Figure 4). This protein contains two Tg1 domains but has no Tg2, Tg3 or ChEL domains (Figure 1-d). Phylogenetic analysis of a large number of sequences [60] classified the urochordates as more closely related to vertebrates than the cephalochordates (amphioxus) and echinoderms (sea urchin). On the basis of these data, we looked for a protein homologous to thyroglobulin in urochordates. Patricolo et al. demonstrated the presence of thyroid hormones and their involvement in metamorphosis in ascidian larvae from the Urochordata [17]. However, the genome of another urochordate, Ciona intestinalis, was found to contain no sequence homologous to thyroglobulin despite the presence of thyroid hormones. These data suggest that ascidians use other precursor proteins for iodotyrosine synthesis [61]. Together, these data suggest that the origins of the thyroglobulin protein lie in the Echinodermata.

We investigated the origin of the Tg1 region domains, by studying the phylogeny of the Tg1 domains in an analysis including the sea urchin protein (Echinodermata), the amphioxus protein (Cephalochordata), and the zebrafish (Teleostei), zebra finch (Aves) and human thyroglobulins. Our results suggest that the second Tg1 domain of the sea urchin protein is the ancestor of the sixth Tg1 domains of the amphioxus protein, while Tg1 domains 1, 2, 3, 4 and 5 of the amphioxus protein probably resulting from the duplication of domain 6. The phylogenetic analysis suggested that the Tg1-1, Tg1-2, Tg1-8 and Tg1-10 domains ofthyroglobulin were derived directly from the Tg1 domains of the amphioxus protein (Figure 2). The separation of thyroglobulin domains into two major branches may indicate two different origins of thyroglobulin Tg1 domains. The thyroglobulin Tg1 domains clustering with the amphioxus protein Tg1 domains are located at the end of the Tg1 region. We suggest that the thyroglobulin Tg1 domainsduplicated from the two ends to the center of the Tg1 region. The number of Tg1 domains presence increases with the number of evolutionary steps, suggesting that the evolution of thyroglobulin function may be dependent on number of Tg1 domains. However,the branching of the tree for Tg1 domains has only weak bootstrap support. (Figure 2 and Additional file 6: Figure S2), probably due to the length of time over which evolution has been occurring. Each Tg1 domain is free to evolve by itself, but the overall structure of the Tg1 region is conserved (Figure 3).

### Involvement of the Tg2 region in cell adhesion

The presence of the Tg2 region in the SCUBE protein of many species suggests that these proteins may have a common function. SCUBE is a protein found in many embryonic tissues [62]. In zebrafish, mutations in the SCUBE2 gene are associated principally with developmental deficits [63]. A recent study showed that SCUBE1 was an adhesive molecule mediating platelet-matrix interaction and ristocetin-induced platelet agglutination [64]. On the basis of its secretory nature, SCUBE3 is thought to function locally or at distance, in a paracrine or endocrine fashion [65]. However, the exact functions of SCUBE3 remain elusive. On the basis of these and published results, we suggest the Tg2 region isinvolved in thyroglobulin-mediated cell adhesion. The conservation of the GCC2-GCC3 domain in the Tg2 region highlights the structural conservation of this region. The function of the GCC2-GCC3 domain remains unknown, but this domain is present in the human SVEP1 protein. The functional annotation of this protein indicates a role in cell adhesion. This is potentially consistent with our hypothesis that the Tg2 region is involved in cell adhesion.

### The Tg3 region may have a structural function

Cysteine is important for the correct three-dimensional structure of a protein, through its role in the formation of disulfide bonds. Misfolded proteins are recognized as abnormal and disposed of by a non lysosomal proteolytic pathway. Hishinuma et al [66] showed that replacement of the cysteine residues of (C1236R) (C1995S) thyroglobulin prevent the protein from forming the disulfide bonds required for thyroglobulin monomer production. As a result, intracellular transport is blocked and both these mutated thyroglobulins are retained in the endoplasmic reticulum. The high degree of cysteine residue conservation in Tg3 domains and in Tg3 regions from the 13 species used to generate the phyogenetic tree, from Actinopterygii to humans, highlights the importance of correct disulfide bond formation to the the tertiary structure of thyroglobulin. In a recent study, Targovenik et al [67] reviewed the cysteine mutations in thyroglobulin andshowed that more than half these mutations (55%) occurred in the Tg3 region. They also reported changes to the three-dimensional structure of thyroglobulin in the presence of cysteine mutations in the Tg3 region. The presence of Tg3 regions only in thyroglobulin proteins may be explained by a structural function, the disulfide bonds being essential to the three-dimensional structure of the molecule. The region of homology highlighted here between the zebrafish Tg3 region and the amphioxus protein suggests that this best conserved region between Tg3 domains may be the origin of these domains. There are two Tg3 subgroups, a and b. We therefore suggest that the original sequence duplicated twice initially, to generate the Tg3-a and Tg3-b domains. The Tg3-a domain duplicated three times, generating Tg3-a1, Tg3-a2 and Tg3-a3, and the Tg3-b domain duplicated twice, giving rise to Tg3-b1 and Tg3-b2.

### The ChEL domain is involved in protein transport

The two studies mentioned above [48,49] demonstrated that a role for the ChEL domain in the dimerization and transport of thyroglobulin. Kim et al [68] indicated that mutations affecting the ChEL domain of mouse thyroglobulin resulted in the synthesis of a full-length thyroglobulin that folded abnormally, preventing its transport to the Golgi complex. However, the ChEL domain is present in structural proteins, as described by Krejci et al [25]. We demonstrated the similarity of this domain between thyroglobulin, esterase and neuroligin proteins, neuroligins being heterophilic cell adhesion proteins [69]. We suggest that the ChEL domain is involved in thyroglobulin transport (thyrocyte to apical membrane) and dimerization, with a possible additional function in cell adhesion. Phylogenetic studies of esterase domains from less evolved species have indicated that the thyroglobulin ChEL and esterase domains have a common ancestor [26]. Additional file 2: Tables S2, Additional file 3: Table S3, Additional file 4: Table S4 and Additional file 5:Table S5 show the pairwise distances between whole thyroglobulin protein sequences and each region of thyroglobulin. The ChEL domain is the region of thyroglobulin for which distances were lowest between different species. This suggests that the thyroglobulin ChEL domain may have been less subject to rearrangement during evolution than the other domains.

### Existence of other thyroid hormone synthesis sites

We show here that not all the thyroid hormone synthesis sites characterized to date are systematically present in all species with a thyroglobulin protein. The lack of some thyroid hormone synthesis sites in some more highly evolved species ( 3 in zebra finch, zebrafish and fugu), the presence of only one site in the amphioxus protein and the total absence of thyroid hormone synthesis sites in the sea urchin protein may be explained by the relocation of these sites. Thyroid hormone synthesisrequires tyrosine residue iodination. The sea urchin protein has five tyrosine residues (positions 3, 24, 31, 34 and 102) and at least one of these residues is a thyroid hormone synthesis site. The lack of sites in amphioxus, zebrafinch, zebrafish and fugu may be explained by an absence of need for large thyroid hormone production or the use of other tyrosine residues as thyroid hormone synthesis sites.

We explored the function of thyroglobulin by phylogeny; we compared the thyroglobulin regions of echinoderms and vertebrate species. Our results suggest that the Tg1 region may have been the first to appear in the thyroglobulin protein. The Tg1 regionwas also subject to the largest number of rearrangements during evolution. The Tg2, Tg3 and ChEL regions are present only in the thyroglobulin of vertebrates, suggesting a link between these regions and an adaptive function of thyroglobulin. The thyroglobulin protein seems to result from the assembly of the four regions. We found no precursor of thyroid hormones with only two or three of these regions in databases. We therefore suggest that the Tg2, Tg3 and ChEL regions appeared in thyroglobulin at the same time. These data support the hypothesis of potential additional functions of thyroglobulin in the cell, as an iodine reservoir, in cell-cell adhesion and in binding. As each thyroglobulin region may have a specific function in the protein, a mutation in one region may have consequences for the specific function of this region, resulting in a different pattern of phenotypic expression.

## Note

A recent study raised the question of human DNA contamination in genomic databases [70], The first 5477 bp of chromosome 11 in zebrafish is 100% identical to human chromosome 4. We verified the zebrafish thyroglobulin located on chromosome 16 at position chr16:33,835,318-33,852,335, and the human thyroglobulin located on chromosome 8 at position chr8:133,909,894-134,147,141.

## Competing interests

The authors declare that they have no competing interests.

## Authors’ contributions

YM AB conceived the analysis. CJ FS contributed to discussion and edited the manuscript. AB analyzed data and wrote the manuscript. All authors read and approved the final manuscript.

## Supplementary Material

Additional file 1 **Figure S1.** The phylogenetic analysis of Tg1 domains from the thyroglobulins of 13 species. The phylogenetic analysis of thyroglobulin Tg1 domains from 13 species (human, marmoset, rat, mouse, panda, dog, horse, pig, cow, opossum, zebra finch, zebrafish and fugu) and the Tg1 domains of thyroglobulin-like proteins from Ciona intestinalis, amphioxus and sea urchin. Evolutionary history was inferred by the neighbor-joining method. The bootstrap consensus tree inferred from 1000 replicates is taken to represent the evolutionary history of the taxa analyzed. The numbers at nodes representing bootstrap scores. Click here for file

Additional file 2 **Table S1.** Estimation of evolutionary divergence between the thyroglobulin protein sequences of 13 species + the thryoglobulin-like sequences of Ciona intestinalis, amphioxus and sea urchin. The number of amino-acid substitutions per site between sequences is shown. Standard error estimates are shown above the diagonal and were obtained by a bootstrap procedure (10000 replicates). Analyses were conducted with the Jones-Taylor-Thornton matrix-based model. The rate variation between sites was modeled with a gamma distribution (shape parameter = 1). Click here for file

Additional file 3 **Table S2.** Estimation of evolutionary divergence between the Tg1 region sequences of thyroglobulins from 13 species. The number of amino acid substitutions per site between sequences is shown. Standard error estimates are shown above the diagonal and were obtained by a bootstrap procedure (10000 replicates). Analyses were conducted with the Jones-Taylor-Thornton matrix-based model. The rate variation between sites was modeled with a gamma distribution (shape parameter = 1). Click here for file

Additional file 4 **Table S3.** Estimation of evolutionary divergence between the Tg3 region sequences of thyroglobulins from 13 species. The number of amino acid substitutions per site between sequences is shown. Standard error estimates are shown above the diagonal and were obtained by a bootstrap procedure (10000 replicates). Analyses were conducted with the Jones-Taylor-Thornton matrix-based model. The rate variation between sites was modeled with a gamma distribution (shape parameter = 1). Click here for file

Additional file 5 **Table S4.** Estimation of evolutionary divergence between the ChEL region sequences of thyroglobulins from 13 species. The number of amino acid substitutions per site between sequences is shown. Standard error estimates are shown above the diagonal and were obtained by a bootstrap procedure (10000 replicates). Analyses were conducted with the Jones-Taylor-Thornton matrix-based model. The rate variation between sites was modeled with a gamma distribution (shape parameter = 1). Click here for file

Additional file 6 **Figure S2.** ClustalX sequence alignment for thyroglobulins from 13 species. The ClustalX sequence alignment of thyroglobulins from 13 species (human, marmoset, rat, mouse, panda, dog, horse, pig, cow, opossum, zebra finch, zebrafish and fugu) and the amphioxus and sea urchin thyroglobulin-like proteins. Red: the four humanthyroid hormone synthesis sites; in green, the 10 human Tg1 domains; in yellow, the human Tg2 region; in blue, the 5 human Tg3 domains. Click here for file
