# Supplementary figures and images for: Phylogenetic analysis of the human thyroglobulin regions
Source: Thyroid Res. 2012 May 1;5:3. doi: 10.1186/1756-6614-5-3 (PMC3464141; doi:10.1186/1756-6614-5-3)

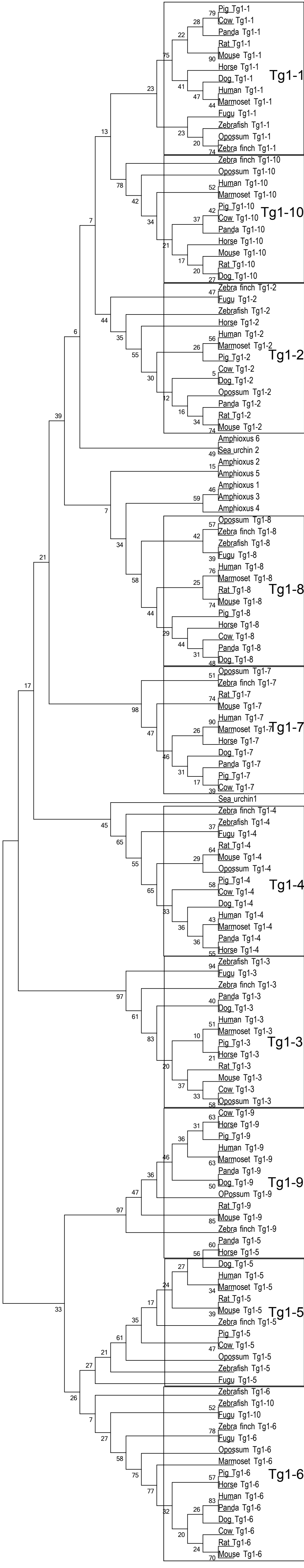

Supplement: Additional file 1 — Figure S1. The phylogenetic analysis of Tg1 domains from the thyroglobulins of 13 species. The phylogenetic analysis of thyroglobulin Tg1 domains from 13 species (human, marmoset, rat, mouse, panda, dog, horse, pig, cow, opossum, zebra finch, zebrafish and fugu) and the Tg1 domains of thyroglobulin-like proteins from Ciona intestinalis, amphioxus and sea urchin. Evolutionary history was inferred by the neighbor-joining method. The bootstrap consensus tree inferred from 1000 replicates is taken to represent the evolutionary history of the taxa analyzed. The numbers at nodes representing bootstrap scores. [file 1756-6614-5-3-S1.pdf]
