# Supplementary material for: Phylogenetic analysis of the human thyroglobulin regions
Source: Thyroid Res. 2012 May 1;5:3. doi: 10.1186/1756-6614-5-3 (PMC3464141; doi:10.1186/1756-6614-5-3)
Supplement: Additional file 2 — Table S1. Estimation of evolutionary divergence between the thyroglobulin protein sequences of 13 species + the thryoglobulin-like sequences of Ciona intestinalis, amphioxus and sea urchin. The number of amino-acid substitutions per site between sequences is shown. Standard error estimates are shown above the diagonal and were obtained by a bootstrap procedure (10000 replicates). Analyses were conducted with the Jones-Taylor-Thornton matrix-based model. The rate variation between sites was modeled with a gamma distribution (shape parameter = 1). [file 1756-6614-5-3-S2.pdf]

	Human	Marmoset	Rat	Mouse	Horse	Panda	Dog	Pig	Cow	Opossum	Zebra finch	Zebrafish	Fugu	C.intestinalis	Amphioxus	Sea urchin
Human		[0.007611]	[0.017347]	[0.015973]	[0.012909]	[0.011286]	[0.014060]	[0.014863]	[0.011674]	[0.024733]	[0.037656]	[0.062244]	[0.086913]	[1.406151]	[2.183744]	[0.571309]
Marmoset	0.112009		[0.018986]	[0.017503]	[0.013608]	[0.013355]	[0.015658]	[0.016485]	[0.013757]	[0.025175]	[0.038138]	[0.064336]	[0.092909]	[1.240063]	[2.005186]	[0.547306]
Rat	0.377192	0.414261		[0.007382]	[0.018656]	[0.018648]	[0.019304]	[0.020689]	[0.016813]	[0.028671]	[0.040939]	[0.068260]	[0.086904]	[1.257586]	[2.627785]	[0.577041]
Mouse	0.357217	0.390880	0.103040		[0.017356]	[0.016152]	[0.017298]	[0.019386]	[0.015723]	[0.028922]	[0.042680]	[0.066139]	[0.085119]	[1.320578]	[2.459525]	[0.609889]
Horse	0.266002	0.293526	0.427825	0.399355		[0.010189]	[0.011705]	[0.011788]	[0.011565]	[0.024036]	[0.036622]	[0.063186]	[0.087682]	[1.294131]	[2.229055]	[0.565820]
Panda	0.271374	0.300503	0.434896	0.410505	0.213528		[0.008956]	[0.012268]	[0.012633]	[0.024900]	[0.037277]	[0.063230]	[0.088036]	[1.382883]	[1.972692]	[0.533108]
Dog	0.281211	0.313861	0.447513	0.424413	0.220188	0.153522		[0.014051]	[0.013243]	[0.024398]	[0.034293]	[0.059977]	[0.088280]	[1.341740]	[2.048369]	[0.539029]
Pig	0.322726	0.345067	0.474364	0.454980	0.260016	0.284509	0.298936		[0.009473]	[0.027944]	[0.040002]	[0.064703]	[0.091283]	[1.388102]	[2.264996]	[0.621159]
Cow	0.299281	0.317168	0.430805	0.406123	0.240507	0.267840	0.275890	0.224553		[0.024379]	[0.036972]	[0.063764]	[0.085067]	[1.186635]	[1.877624]	[0.549576]
Opossum	0.638496	0.661732	0.741269	0.726439	0.642810	0.664362	0.663072	0.709624	0.667708		[0.034668]	[0.066367]	[0.089210]	[1.442755]	[1.987607]	[0.609765]
Zebra finch	0.952762	0.975563	1.054782	1.031108	0.961541	0.977184	0.990116	1.001261	0.960570	0.888810		[0.063936]	[0.084126]	[1.579845]	[2.507270]	[0.508043]
Zebrafish	1.595894	1.659855	1.743004	1.699544	1.604042	1.644867	1.624531	1.669445	1.629906	1.581515	1.522938		[0.057036]	[2.167328]	[2.076955]	[0.687143]
Fugu	2.074401	2.119814	2.137776	2.138405	2.111683	2.135814	2.154558	2.127495	2.045847	2.133502	1.943284	1.461816		[2.094662]	[2.794597]	[0.598314]
C.intestinalis	11.836881	11.352050	11.010537	11.376454	11.332266	11.543390	11.378272	11.817380	11.199215	11.962974	12.344837	15.192344	15.764006		[2.691111]	[13.643540]
Amphioxus	19.023097	18.302167	20.722767	20.611852	19.367899	18.574551	18.503626	19.321095	18.184735	18.551285	20.261977	18.779269	21.009011	18.564030		[11.184003]
Sea urchin	3.120950	2.947310	3.126968	3.192709	2.984354	2.962699	2.847679	3.093708	3.050308	3.187572	2.648947	3.674318	3.258729	18.815198	16.738553	
