# Supplementary material for: Phylogenetic analysis of the human thyroglobulin regions
Source: Thyroid Res. 2012 May 1;5:3. doi: 10.1186/1756-6614-5-3 (PMC3464141; doi:10.1186/1756-6614-5-3)
Supplement: Additional file 4 — Table S3. Estimation of evolutionary divergence between the Tg3 region sequences of thyroglobulins from 13 species. The number of amino acid substitutions per site between sequences is shown. Standard error estimates are shown above the diagonal and were obtained by a bootstrap procedure (10000 replicates). Analyses were conducted with the Jones-Taylor-Thornton matrix-based model. The rate variation between sites was modeled with a gamma distribution (shape parameter = 1). [file 1756-6614-5-3-S4.pdf]

	Human	Marmoset	Rat	Mouse	Horse	Panda	Dog	Pig	Cow	Opossum	Zebra finch	Zebrafish	Fugu
Human		[0.016309]	[0.036383]	[0.032775]	[0.032766]	[0.027021]	[0.027632]	[0.030876]	[0.028374]	[0.067115]	[0.097551]	[0.138733]	[0.183550]
Marmoset	0.124636		[0.039119]	[0.036082]	[0.035707]	[0.029383]	[0.029602]	[0.030700]	[0.030425]	[0.070844]	[0.099590]	[0.143392]	[0.183036]
Rat	0.449229	0.488228		[0.016059]	[0.044616]	[0.039205]	[0.038542]	[0.040018]	[0.037575]	[0.078790]	[0.090870]	[0.150332]	[0.192066]
Mouse	0.382475	0.426391	0.128356		[0.041297]	[0.034778]	[0.036378]	[0.039863]	[0.036326]	[0.075518]	[0.092681]	[0.152850]	[0.195628]
Horse	0.290482	0.334660	0.476990	0.393845		[0.027542]	[0.024645]	[0.028767]	[0.028135]	[0.081039]	[0.108086]	[0.139799]	[0.206830]
Panda	0.279838	0.307077	0.496114	0.411119	0.238251		[0.020236]	[0.027911]	[0.027680]	[0.071096]	[0.100039]	[0.153580]	[0.180684]
Dog	0.290556	0.323797	0.498166	0.424726	0.221174	0.181532		[0.028117]	[0.026639]	[0.069053]	[0.092258]	[0.139890]	[0.172102]
Pig	0.334801	0.351645	0.487120	0.446610	0.268018	0.297161	0.308972		[0.024134]	[0.074031]	[0.100001]	[0.155153]	[0.204219]
Cow	0.302755	0.320908	0.463591	0.408247	0.245683	0.281802	0.278817	0.220215		[0.070064]	[0.097463]	[0.148467]	[0.189014]
Opossum	0.843964	0.885448	0.991852	0.946050	0.949158	0.950755	0.890997	0.947109	0.905723		[0.099267]	[0.163118]	[0.212090]
Zebra finch	1.285588	1.275363	1.230458	1.199707	1.239780	1.264322	1.204174	1.289284	1.224434	1.264075		[0.126929]	[0.160111]
Zebrafish	1.690680	1.742134	1.867547	1.836328	1.570839	1.848856	1.765797	1.883530	1.781377	1.942431	1.605885		[0.091425]
Fugu	2.134041	2.124378	2.235806	2.266487	2.241034	2.186576	2.120403	2.352762	2.190312	2.454529	1.964061	1.213615	
