# Supplementary material for: Phylogenetic analysis of the human thyroglobulin regions
Source: Thyroid Res. 2012 May 1;5:3. doi: 10.1186/1756-6614-5-3 (PMC3464141; doi:10.1186/1756-6614-5-3)
Supplement: Additional file 6 — Figure S2. ClustalX sequence alignment for thyroglobulins from 13 species. The ClustalX sequence alignment of thyroglobulins from 13 species (human, marmoset, rat, mouse, panda, dog, horse, pig, cow, opossum, zebra finch, zebrafish and fugu) and the amphioxus and sea urchin thyroglobulin-like proteins. Red: the four humanthyroid hormone synthesis sites; in green, the 10 human Tg1 domains; in yellow, the human Tg2 region; in blue, the 5 human Tg3 domains. [file 1756-6614-5-3-S6.pdf]

```
Zebrafish       -KISEYQLETES--LSQCEQLRSVSAEQERQHVPQCFEDGRFRHVQCNRGGGECWCVNAE 57
Fugu            -VFYEYSLQSDS--LSPCELLRSEAVAKQQGNVPQCAEDGKFRPLQCSMGGQECWCVDGD 57
Rat             -NIFEYQVDAQP--LRPCELQREKAFLKQDEYVPQCSEDGSFQTVQCQNDGQSCWCVDSD 57
Mouse           -NIFEYQVDAQP--LRPCELQREKAFLKQAEYVPQCSEDGSFQTVQCQNDGQSCWCVDSD 57
Human           -NIFEYQVDAQP--LRPCELQRETAFLKQADYVPQCAEDGSFQTVQCQNDGRSCWCVGAN 57
Marmoset        -NIFEYQVDAQP--LRPCELQRERAFLKQADYVPQCTEDGSFQTVQCQNDGRSCWCVGVD 57
Pig             -NIFEYQVDAQP--LRPCELQRERAFLKRADYVPQCAEDGSFQTVQCKKDGGSCWCVDAD 57
Cow             -NIFEYQVDAQP--LRPCELQRERAFLKREDYVPQCAEDGSFQTVQCGKDGASCWCVDAD 57
Panda           -NIFEYQVEAQP--LRPCELQREEAFLKGADHVPQCTEDGSYQTVQCRNDGGSCWCVDAN 57
Dog             -NIFEYQVDAQP--LRPCELQRERAFLKGAEYVPQCAEDGSYQTVQCQNDGRTCWCVGAD 57
Horse           -NIFEYQVDAQP--LRPCELQRERAFLTRADYVPQCAEDGSFQTVQCGDGGRACWCVGAD 57
Opossum         -NIFEYEVDSQP--LRPCELQREQAFLKKEDHIPQCLADGRFRNVQCSNNGLSCWCVDAE 57
Zebra finch     -NIFEYQTDSQP--LRPCELQREEAFAAGEAYVPQCTEDGQFRTVQCSRNGLSCWCVDEN 57
Sea urchin      -RQYGTHN------ISCHAL-VEQTTANDSAYFPRCTGYNTYSAKQCDSSG-SCWCVGPD 51
Amphioxus       -IEDEFQCGNGR-CIPDSFECDGEDDCRDNSDEVNC-TGCRRGKFMCEDGGCLPSIFRCD 57
                    :.        :                   .*          *  .*     .  :

Zebrafish       GFEIPGSRQNGSSVYCLTTCQLQRQR------ALQTEDATLVPVCLDSGEYQQVQCDASR 111
Fugu            GQEVPGTRTNGSAPLCETNNCYSHHAPDGVCTGVSPCQLQSLLKCSPSGLFEEVQCDHSR 117
Rat             GTEVPGSRQLGRPTACLSFCQLHKQR-ILLSSYINSTDALYLPQCQDSGNYAPVQCDLQQ 116
Mouse           GREVPGSRQLGRPTVCLSFCQLHKQR-ILLGSYINSTDALYLPQCQDSGNYAPVQCDLQR 116
Human           GSEVLGSRQPGRPVACLSFCQLQKQQ-ILLSGYINSTDTSYLPQCQDSGDYAPVQCDVQQ 116
Marmoset        GGEVLGSRQPGRPVACLSFCQLQKQQ-ILLSGYINSTATSYLPQCQDSGDYTPVQCDVRQ 116
Pig             GREVPGSRQPGRPVACLSFCQLQKQQ-ILLSSYINSTATSYLPQCQDSGAYAPVQCDVRR 116
Cow             GREVPGSRQPGRPAACLSFCQLQKQQ-ILLSSYINSTATSYLPQCQDSGDYSPVQCDLRR 116
Panda           GREVPGSRRPARPAACPSFCQLQKQQ-ILLSGYLNGTATAYLPQCQDSGDYAPVQCDLGR 116
Dog             GVEVPGSRQPARPAACLSFCQLQRQQ-ILLSGYLNSTATSYLPQCQESGGYAPVQCDLWQ 116
Horse           GLEVPGSRQPGRPAACLSFCQLQRQR-ILLDGHANSSATSYLPQCEDSGDYAPVQCDIRG 116
Opossum         GTEVPGSKQAGLPDACLSFCQLHKQK-ILVSSYINSTATSYLPQCQDSGEYEPVQCDLAR 116
Zebra finch     GVEVPGSKQNGYPISCLSFCQLQRQR-ILVSRYINSSSISYIPQCLDSGAFDEVQCDMEL 116
Sea urchin      G--VHGADR-------------------------------------------EMEADRTK 66
Amphioxus       GIGDCDDDSDETNCRLGFLDTLGGVP-----DQLTTTTQTVPRPYPVTPIPPSSILEQGL 112
                *    .                                                  :

Zebrafish       SQCWCVDLEGMEIYGTRQNGKPSKCPGTCEVRERRLLHGIGERSPPQ----CSDDGGFLP 167
Fugu            GQCWCVDQNGMELYGTRQNGRPSRCPGSCEVRSRSLLHSSTPHTPPQ----CDGSGSFLP 173
Rat             VQCWCVDTEGMEVYGTRQQGRPTRCPRSCEIRSRRLLHGVGDKSPPQ----CDADGEFMP 172
Mouse           VQCWCVDTEGMEVYGTRQQGRPTRCPRSCEIRNRRLLHGVGDRSPPQ----CTADGEFMP 172
Human           VQCWCVDAEGMEVYGTRQLGRPKRCPRSCEIRNRRLLHGVGDKSPPQ----CSAEGEFMP 172
Marmoset        VQCWCVDAEGMEVYGTRQLGRPRRCPRSCEIRNRRLLHGVGDKSPPQ----CSAEGEFMP 172
Pig             EQCWCVDAEGMEVYGTRRLGRPARCPGCCEIRNRRLLHGVGDKSPPQ----CSADGTFLP 172
Cow             RQCWCVDAEGMEVYGTRQQGRPARCPRSCEIRNRRLLHGVGDRSPPQ----CSPDGAFRP 172
Panda           EQCWCVDTEGMEVYGTRQRGRPTRCPRSCEIRNRRLLHGVGDKSPPQ----CSPDGGFLP 172
Dog             QQCWCVDAEGMEVYGTRQLGRPARCPRSCDIRNRRLLHGVGDKSPPQ----CSPDGAFMP 172
Horse           MQCWCVDADGMEVYGTRQLGRPTRCPRSCEIRNRRLLHGVGEKSPPQ----CSPEGEFLP 172
Opossum         VQCWCVDSEGMEVYGTRQQGKPTRCPGSCEIRNRRILHGVGEKSPPQ----CSSDGEFMP 172
Zebra finch     GQCWCVDPEGMEIYGTRQKGKPAQCPGNCEIRDRRILHGFGEKSPPQ----CSADGEFLP 172
Sea urchin      ---LCVEAP----------------CRQELESNQDKVNLGQNLTSNPV----CDPQGMYIP 104
Amphioxus       LLAVCTEQEFTCADGSKCVPRLFLCDGEKDCSDNSDEQDCAPRTCSEDKFTCSSGDCIPK 172
                    *.:                 *    :  .     .    : .     *   .

Zebrafish       VQCKFVNTTDRMIFDLLHTFNRQPEVFQTFSGFRKAYPELSSYCFCADSRGREMPSTGVE 227
Fugu            VQCVFINTTTGTQLDMMTVFNSFPEAFETFSGFRKLFPTVSSYCFCSDSRGREMHNTGVE 233
Rat             VQCKFVNTTDMMIFDLIHNYNRFPDAFVTFSAFRNRFPEVSGYCYCADSQGRELAETGLE 232
Mouse           VQCKFVNTTDMMIFDLIHNYNRFPDAFVTFSSFRGRFPEVSGYCYCADSQGRELAETGLE 232
Human           VQCKFVNTTDMMIFDLVHSYNRFPDAFVTFSSFQRRFPEVSGYCHCADSQGRELAETGLE 232
Marmoset        VQCKFVNTTDMMIFDLVHNYNRFPDAFVTFSSFQSRFPEVSGYCHCADSQGRELAETGLE 232
Pig             VQCKFVNTTDMMLFDLVHSYNRFPDAFVTFSSFRSRFPEVSGYCHCADSQGRELAETGLE 232
Cow             VQCKLVNTTDMMIFDLVHSYSRFPDAFVTFSSFRSRFPEVSGYCYCADSQGRELAETGLE 232
Panda           VQCKFVNTTDMMIFDLVQSYNRFPDAFVTFSAFRSKFPEVSGYCHCADSQGRELAETGLE 232
Dog             VQCKFVNTTDMMIFDLVHSYNRFPDAFVTFSAFRSKFPDVSGYCHCADSLGRELAGTGLE 232
Horse           VQCKFVNTTDMMIFDLVHSYNRFPDAFVTFSAFRSRFPEVSGYCHCADSQGRELAETGLE 232
Opossum         VQCKFVNTTDMMIFDLVNSYNRFPEAFLTFSSFRSRFPEVSGYCYCADSQGRELAETGLE 232
Zebra finch     VQCKFVNTTDMSVLDLVHSYNRLPRAFQKFSSVREVFPEISGYCYCVDSLGRELADTGLE 232
Sea urchin      QQCTADGT---------------------------------------------------- 112
Amphioxus       AWTCDLDVDCADGSDETEELCQETNCTEKEYRCQNSVKCIHLTWFCDGDKDCREGEDEAD 232
                      ..
```

```
Zebrafish      LLLDEVYDTAFSDQTAGSSFAESNMYRILNRRYLAV---QLAINGKFRCPSPCESERAAA 284
Fugu           LLLSDVYDSAFDAHPPVRSFAQSNIYRVLQRRMLAV---RLRITGRFRCPSPCEEERWAA 290
Rat            LLLDEIYDTIFAGLDQASTFTQSTMYRILQRRFLAI---QLVISGRFRCPTKCEVEQFTA 289
Mouse          LLLDEIYDTIFAGLDQASTFTQSTMYRILQRRFLAI---QLVISGRFRCPTKCEVEQFAA 289
Human          LLLDEIYDTIFAGLDLPSTFTETTLYRILQRRFLAV---QSVISGRFRCPTKCEVERFTA 289
Marmoset       LLLDEIYDTIFAGLDLASTFTETTLYRILQRRFLAV---QSLISGRFRCPTKCEVERFTA 289
Pig            LLLDEIYDTVFAGLDLASSFTETTLYRILQRRFLAV---QLVTSGRFRCPTKCEVERFAA 289
Cow            LLLDEIYDTIFAGLDLASTFAETTLYRILQRRFLAV---QLVISGRFRCPTKCEVERFAA 289
Panda          LLLDEIYDTIFAGLDLASTFAESTLLRILQRRFLAV---QLVVSGRFRCPTKCEVERFTA 289
Dog            LLLDEIYDTIFAGLDLASTFAETTLYRILQRRFLAV---QLVLSGRFRCPTRCEVEQFTA 289
Horse          LLLDEIYDTIFAGLDLPSTFSETTLYRILQRRFLAV---QLVISGRFRCPTKCEVERFTA 289
Opossum        LLLDEIYDTIFAGLDPASTFTETTLYRILQRRFLAV---QLVISGRFRCPTKCEVERLAA 289
Zebra finch    LLLDEVYETIFSEPEPARTFTESSIHRILQRRFLGV---QLATSGKFRCPSRCEAERAAA 289
Sea urchin     ------------------------------------------------------------
Amphioxus      CDQQESMACSDGEWRCDAGKCISAMWRCDGEQDCMDGTDEEFCHIRLRAQNLTSLDIPTP 292

Zebrafish      SQAGNSFVPSCTDNGAYVPTQCQSGGQCWCVDTYGKEIFGTRQNGVPKCSTGVKDCLSER 344
Fugu           TEAHSVYIPSCEPGGAFTTRQCQQGGQCWCVDHTGQELPGTRQQALLDCKLGPDSCPARR 350
Rat            TSFGHPYIPSCHRDGHYQTVQCQMERMCWCVDAQGIEIPGTRQQGQPLFCAKDQSCASER 349
Mouse          TRFGHSYIPRCHRDGHYQTVQCQTEGMCWCVDAQGREVPGTRQQGQPPSCAADQSCALER 349
Human          TSFGHPYVPSCRRNGDYQAVQCQTEGPCWCVDAQGKEMHGTRQQGEPPSCAEGQSCASER 349
Marmoset       TSFGHPYVPSCRRNGDYQAVQCQKEGPCWCVDAQGKEIHGTRQQGEPPSCAEGQSCVSKR 349
Pig            TSFGHPYVPSCGRDGEYQAGQCQQEGPCWCVDAQGQEIPGTRRPSEPLSCAKGQSCPLER 349
Cow            TSFRHPYVPSCHPDGEYQAAQCQQGGPCWCVDSRGQEIPGTRQRGEPPSCAEDQSCPSER 349
Panda          SSFGHPYIPSCRRNGGYQAVQCQRGGPCWCADAQGKEIHGTRRQGERPSCAEDQFCASER 349
Dog            SSFGHPYTPSCRRDGGFQAVQCQRGGPCWCVDARGKEIPGTRRPGERPSCAETQLCASER 349
Horse          SSFGHPYVPSCHRNGDYQAVQCQREGPCWCADAQGKEIYGTRRQGEPPSCAEDQSCTSER 349
Opossum        ISFQHSYMPSCKDNGEYQPVQCQKERLCWCVDAKGKEISGTRQQGKLPSCGAERSCVSER 349
Zebra finch    QRFQQPLVPSCAADGGHTSLQCHPWGQCWCVDTQGHEVPGTRRRGQPLACGEEQSCISER 349
Sea urchin     --------------------------QCWCSEPDGTMIPNTKR-----ASGESVTC---- 137
Amphioxus      SNLTLGGNDTGDHVCALITKMLPYFRQHEIKPRCNTVTVGIYISSITRLIKEPVKPLSTR 352
                                                 .    .

Zebrafish      RQALSKLFYGPAGDFSKSNVFSS------SKDTVSFLGTCSPEFQELLANSGLLQSLPEL 398
Fugu           HRALFHLLSGSVAVPLQTSISDR------SQTSCNSL---LQTLRDLLP--------VEV 393
Rat            QQALSRLYFETPGYFSPQDLLSS-EDRLVPVSGARLDISCPPRIKELFVDSGLLRSIAVE 408
Mouse          QQALSRFYFETPDYFSPQDLLSS-EDRLAPVSGVRSDTSCPPRIKELFVDSGLLRSIAVE 408
Human          QQALSRLYFGTSGYFSQHDLFSSPEKRWASPRVARFATSCPPTIKELFVDSGLLRPMVEG 409
Marmoset       QQALSRLYFGTSGYFSRHDLFSSWEEGRASPRVARFATPCPPTIKELFVDSGLLHPMVEG 409
Pig            RRALSRLHLGPSGYSGQRGSFLAAERAPVSQTVPSFAASCPLPLKELFVESGILQPVVQG 409
Cow            RRAFSRLRFGPSGYFSRRSLLLAPEEGPVSQRFARFTASCPPSIKELFLDSGIFQPMLQG 409
Panda          RQALSRLHFGPSGYFSQHRLFLAPEGRQVSPRVARFATSCPPLIKELFVDSGILHPMVEG 409
Dog            RQALSRLFFGPSGYFSQHSLFLAPDGRQVSQRVARFATSCPPLIKELFVDSGILRPMVEG 409
Horse          RQALSRLHFGPSGYFSRHSLFLAPEKRQVSQRVARLATSCPLLIKELFVDSGILQPMVEG 409
Opossum        QQALSKLFFGPGGHFSQHSLFFAPEERQEPQKVARFAKTCPPFFKELFVDSGLLLPFTER 409
Zebra finch    RRALSRLLYGPAGYFSQSSLFSTPDKQS--DKTDGFSRPCPPSFKELFLDSGLSSPLAQS 407
Sea urchin     ------------------------------------------------------------
Amphioxus      FVIPEVGQFDKPGVCPQLGFPLHQSSPTCSRDACVSDFHCPGDLKCCVNGCGGTMCVDPS 412

Zebrafish      ER----PKVTN--------ILADVLQGMFPSGALALKALALAQNPKRLQENLFGGKFLKN 446
Fugu           EF----PPFLS--------QLVEVVDGLFRTVGGALRALSHS-SPQRLNENLFGGKFLKE 440
Rat            RYQQLSESRS---------LLREAIRAIFPSRELAGLALQFTTNPKRLQQNLFGGTFLVN 459
Mouse          HYQRLSESRS---------LLREAIRAVFPSRELAGLALQFTTNPKRLQQNLFGGTFLAN 459
Human          QSQQFSVSEN---------LLKEAIRAIFPSRGLARLALQFTTNPKRLQQNLFGGKFLVN 460
Marmoset       QSQRFSVSES---------LLKEAIRAIFPSRELAHLALQFTTNPERLQQNLFGGKFLVN 460
Pig            QKKEVTAATES--------LLKEGLRGIFPSQELARLALQFTANPKRLQQNLFGGRFLAN 461
Cow            RDTRFVAPES----------LKEAIRGLFPSRELARLALQFTTNAKRLQQNLFGGRFLVK 459
Panda          QDQQFSASET---------LLREAIGAIFPSRELARLALQFTTDPKRLQQNLFGGKFLVN 460
Dog            QDKQFSASET---------LIREAIGAIFPSRELARLALQFTTDPTRLQQNLFGGKFLVN 460
Horse          LDKQFSASES---------LLKEAIRVIFPSRELARLALQFTTNPKRLQQNLFGGKFLVN 460
Opossum        EGATQFFTLET--------FLSEAFGVLFPSRDLALLALQFTTDPKRLQQNLFGGKFLEN 461
Zebra finch    PFASQTPELET--------ILSEAITGMFPSRELARVALQFTANPKRFQENLFGGRFLKN 459
Sea urchin     ------------------------------------------------------------
Amphioxus      PEPTTLFEQEAGCANYDCGMQGAYCSGIVAGSAQCYCRPRSECEAKDTVCDKYGTMYPSM 472
```

```
Zebrafish       AGNFNFSGTVGDSGTLSFRQIFSQVGLTQV-----VTDLMQLAK----------TFTDSS 491
Fugu            IASSNISGVLGSQGTF--------------------VRDWVS------------------ 462
Rat             AAQLNLSGALGTRSTFNFSQFFQQFGLPGFLVRDRATDLAKLLPVSLDSSPTPVPLRVPE 519
Mouse           AAQFNLSGALGTRSTFNFSQFFQQFGLPGFLNRDRVTTLAKLLPVRLDSSSTPETLRVSE 519
Human           VGQFNLSGALGTRGTFNFSQFFQQLGLASFLNGGRQEDLAKPLSVGLDSNSSTGTPEAAK 520
Marmoset        VGQFNLSGALGTRGTFNFSQFFQQLGLASFSNGGRLEDFAKPLSVELDSNSSAETPEASK 520
Pig             VGQFNLSGALGTRGTFNFSHFFQQLGLPGFQKRQALADPAKSLSVGLDSNPATEAPEALK 521
Cow             VGQFNLSGALGTRGTFNFSHFFQQLGLPGFQDGRALADLAKPLSVGLNSNPASEAPKASK 519
Panda           VGQFNLSGALGTKGTFNFSQFFQQFGLPGFQNGG--TNLSKPLSLGFDAGPATEPPEASK 518
Dog             VGRFNLSGAIGTRGTFNFSQFFQQFGLPGFQNSE--TDLAQPLSVGLDSQPATAPPEASK 518
Horse           AGQFNLSGALGTRGTFNFSQFFQQFGLPGFQNRGPPADRAEPLSLGLDSSPATESPEASK 520
Opossum         ISRFNLSGAIGARSTFNFTQFFQQIGLQGIKNRGDLVELTQQLSAGPD-----EVLANSK 516
Zebra finch     LIQFNFTGVLGTNGKYSIGQFFPQEDLSEMGNGQGPLESAEAFS-----------LEASK 508
Sea urchin      ------------------------------------------------------------
Amphioxus       CHFEAFACEWGFPMEPAPCQDYNVQDIFKCKNGIRFVLKPYICDG--------------I 518

Zebrafish       ENLNLDQEISDAFGRSVNLKNNREMIKLVSMALENTHFLSTLRVAIKQLKAEETTQLGSL 551
Fugu            -------------GKGDSLQENRVLVESVRRVLGDPAFLSSLKVALQGHSSLLPPEQ--I 507
Rat             KRVAMNKSVVGTFGFKVNLQENQDALKFLVSLMELPEFLVFLQRAVS-VPEDRARDLGDV 578
Mouse           KTVAMNKRVVGNFGFKVNLQENQDALKFLVSLLELPEFLVFLQRAIS-VPEDIARDLGDV 578
Human           KDGTMNKPTVGSFGFEINLQENQNALKFLASLLELPEFLLFLQHAIS-VPEDVARDLGDV 579
Marmoset        KAVAMNKSTVGSFGFEINLQENQNALKFLSSLLELPEFLLFLQHAIS-VPEDVARDLGDV 579
Pig             MGVAMNKTVVGSFGFEVNLQENRNALTFLSSLLELPEFLLFLQHAIS-VPEDIARDLGDV 580
Cow             IDVALRKPVVGSFGFEVNLQENQNALQFLSSFLELPEFLLFLQHAIS-VPEDIARDLGDV 578
Panda           KGAATNRMIVGSFGFEINLQENQNALTFLSSLLELPEFLLFLQQALS-VPEDIARDLGDV 577
Dog             EGTAMNVPVVGSFGFQINLQENQNALTFLASLLELPEFLLFLQHAIS-VPEDIAKDLGDV 577
Horse           KGVAMNKPVVGSFGFEINLQENLNALKFLVSLLELPEFLLFLQQAIS-VPEDIARDLGDV 579
Opossum         ERLTLNQSIVGSLGQKVNLQDNQNKLKFLSSIMELPEFLLFLKGVIS-VPENIARSLGDV 575
Zebra finch     GSSILSKPLVSSFVGTVTLQDNQNLMKFLSYVLELPEFFTFLQQVIS-VPESVAEDLGEV 567
Sea urchin      ------------------------------------------------------------
Amphioxus       TDCFDGSDETGCPFKEPPPTSPTATVCNLPLYRGRPCADATSRLRWYYNSQKRTCLTYTH 578

Zebrafish       FRDIFQKSDVCKPMSSSSAPYLPQCTEDGLYQDVQCQGSECWCVDSRGLEVPGSRTTGSR 611
Fugu            LTPLLR--SCSGQDVTKHATFLPRCTPSGAFQEIQCDSGECWCVDSQGHEVLGSRTAGRP 565
Rat             MEMVFSAQACKQ---TSGRFFVPSCTAEGSYEDIQCYAGECWCVNSQGKEVEGSRVSGGH 635
Mouse           MEMVFSAQACKQ---MPGKFFVPSCTAGGSYEDIQCYAGECWCVDSRGKELDGSRVRGGR 635
Human           METVLSSQTCEQ---TPERLFVPSCTTEGSYEDVQCFSGECWCVNSWGKELPGSRVRGGQ 636
Marmoset        MEIVLNSQTCEQ---TPERLFVPSCMAEGSYEAVQCFAGECWCVDSWGKELPGSRVRGGQ 636
Pig             MEMALSSQGCEQ---TPGSLFVPSCTAEGSYEDVQCFAGECWCVDARGRELAGSRARGGR 637
Cow             MEMVFSSQGCGQ---APGSLFVPACTAEGSYEEVQCFAGDCWCVDAQGRELAGSRVRGGR 635
Panda           MEIVLSSQGCEQ---TPGSLFVPRCSAEGSYEDVQCFAGECWCVDSRGKELAGSRVRGAR 634
Dog             MEMVLSSQDCEQ---TPGNLFVPQCSAEGDYEDVQCFAGECWCVDSRGKELAGSRVRGGR 634
Horse           MELVLSSQGCEQ---TPGGLFVPSCTAEGSYEDVQCFAGECWCVDSRGKELPGSRVRGGR 636
Opossum         IKMALESQACEQ---PSRELFVPTCTDGGNYDQVQCFAGECWCVDPQGRELRASRIRGQR 632
Zebra finch     VKLALGSGGCGE---EPRDLFVPTCTKEGRYEEVQCYAGECWCLDTSGKEVPGSRRRGKR 624
Sea urchin      ------------------------------------------------------------
Amphioxus       SGCDRNANSFGTREECLENVNAKRCVVKRTYSQRIPDLPQTTAAPTKDPEVVCNMLKDRG 638

Zebrafish       PRCPSQCEKERQMAIAVKASSSAGSEVFIPKCETDGAYVARQCLGKSCFCVDRSGTKL-- 669
Fugu            SRCPSICERQQWSALNMRKNMAAGAEIYVPVCSEDGDFLPLQCVGSHCFCVDAKGN---- 621
Rat             PRCPTKCEKQRAQMQNLAGAQPAGSSFFVPTCTSEGYFLPVQCFNSECYCVDAEGQVIPG 695
Mouse           PRCPTKCEKQRAQMQSLASAQPAGSSFFVPTCTREGYFLPVQCFNSECYCVDTEGQVIPG 695
Human           PRCPTDCEKQRARMQSLMGSQPAGSTLFVPACTSEGHFLPVQCFNSECYCVDAEGQAIPG 696
Marmoset        PRCPTDCEKQRARMQSLMGSQPAGSSVFVPACTSEGHFLSVQCFKAECYCVDAEGQAIPG 696
Pig             PRCPTACEKQRERMQSLLGRQPAGSSVFVPSCTREGHFLPVQCFSSDCYCVDADGQPIPG 697
Cow             PRCPTECEKQRARMQSLLGSQPAGSSLFVPACTSKGNFLPVQCFNSECYCVDTEGQPIPG 695
Panda           PRCPTECEKQRALMQILSGSLPAGASLFVPSCSSEGHFLPVQCFNSECYCVDAEGQAIPG 694
Dog             PRCPTECEKQRALMRSLSGSLPAGASLFVPSCTSEGHFLPVQRFNSECYCVDTNGQAIPG 694
Horse           PRCPTKCEKQRARMQSLSGSQPAGSSLLVPSCTSEGHFLPVQCFSSECYCVDAEGQPIPG 696
Opossum         PRCPSECEKQREWAQVFMRSLPAGSSLSVPSCSSDGSFLPVQCSDSGCYCVDSEGQTIPG 692
Zebra finch     PKCPTECEKQRRNLKNLKQSLPAGSDLFIPSCTEDGDFLPLQCYGTNCFCVDLNGKTIPG 684
Sea urchin      ------------------------------------------------------------
Amphioxus       PCRGRQKKYYYLSTQRRCVRFTYGGCGGNDNRFHTYMECMDFCNDPRTKCQRQRTKAANA 698
```

```
Zebrafish       GIQSSGSSLQCPTSCQAIAIQQFQSTIRSLLSDPLSVTQLSEIYIPRCSYDGSWHQIQCD 729
Fugu            NVSPAGGAVSCKISIYTIHTKHFTTSHDCKMIVPCSLTGK---YVHPCQDS-------CS 671
Rat             TQSTIGEPKLCPSVCQLQAEQAFLGVVGVLLSNSSMVPPISSVYIPQCSTSGQWMPVQCD 755
Mouse           TQSTVGEAKQCPSVCQLQAEQAFLGVVGVLLSNSSMVPSISNVYIPQCSASGQWRHVQCD 755
Human           TRSAIGKPKKCPTPCQLQSEQAFLRTVQALLSNSSMLPTLSDTYIPQCSTDGQWRQVQCN 756
Marmoset        TQSVMGKPKKCPTPCQLQAEQAFLWTVQALLSNSSMLPTLPDTYIPQCRADGRWRQVQCD 756
Pig             TRTAPGEPKQCPTPCQLQAEQAFLGTVRGLISNPSEPPVLSSIYIPQCSASGQWRRVQCD 757
Cow             TRSALGEPKKCPSPCQLQAERAFLGTVRTLVSNPSTLPALSSIYIPQCSASGQWSPVQCD 755
Panda           TRSVPGEPKPCPTPCQLQAEQAFLGVARALGSGSSMLPSFSSSYVPQCSTSGQWRPVQCD 754
Dog             TRSLPGEPKQCPTPCQLQAEQAFLGIVQVLGSDSSMLPTFSSSYIPQCSTAGQWRPVQCD 754
Horse           TRSLPGEPKQCPTPCQLQAEQAFLGAVRALVSNSSTLPTLSSIYIPQCSANGQWRHVQCD 756
Opossum         TEKMNGESKQCPTTCQLAAEQMFLWTARTLLGEPSLLPQLSDIYIPQCNSRGQWKRVQCN 752
Zebra finch     IKGEAGNPMKCPSACQVAAGQEFLRAVKLLLSDPSAVPELSSLHLPQCDAGGGWRQVQCS 744
Sea urchin      ------------------------------------------------------------
Amphioxus       GLQVGMFITECRHGGFKPTCSRQSDDTGGQGLQTYMFRSVRHMDGQGLQTYMFRSVRHMD 758


Zebrafish       GPPEQAIEFYH------------------------------------------------- 740
Fugu            PSP--------------------------------------------------------- 674
Rat             GPHEQVFEWYERWNTQNSDGQELTTATLLMKLMSYREVASTNFSLFLQSLYDAGQQSIFP 815
Mouse           GPHEQVFEWYERWKTQNGDGQELTPAALLMKIVSYREVASRNFSLFLQSLYDAGQQRIFP 815
Human           GPPEQVFELYQRWEAQNK-GQDLTPAKLLVKIMSYREAASGNFSLFIQSLYEAGQQDVFP 815
Marmoset        GPPEQVFQWYQQWEAQNK-GQELTPAELLVKIVSYREAASGNFSLFIQSLYEAGEQGVFP 815
Pig             GPPEQAFEWYERWGAQSRSGQELTPAELLMKIMSYREAASGSFRVFIQNLYEAGQQGIFP 817
Cow             GPPEQAFEWYERWEAQNSAGQALTPAELLMKIMSYREAASRNFRLFIQNLYEAGQQGIFP 815
Panda           GPPEQAFEWYERWGAQNNSGQELTPAELLEKIMSYREAASGSFRLFIQSLYEAGQQGIFP 814
Dog             GPPEQAFEWHERWEAQNNSGQELTPAELLMKIMSYREAASGSFRLFIQSLYAAGQQGIFP 814
Horse           GPPEQAFEWYERWRAQNNGGQELSPAQLLMKIMGYREAASRSFRLFIQSLYEAGQQGIFP 816
Opossum         GPPEQAFQWYQRWITQNNGGQELPLAEVIQRMMSYKEASSEAFSIFIEKLYEAGHQNIFP 812
Zebra finch     GPPEQAFQWYERWIAENNQGKPLPVPELVNIIAGYKEASSGDFSAFVKALYEAGHQNVFP 804
Sea urchin      ------------------------------------------------------------
Amphioxus       GQGLQTYMFRSVRHTGGQ---------------------------GLQTYMSRSVRHTGG 791


Zebrafish       -------------LLDGNSEAVYGPSVYLNPLSLWRLIRLDDSGYPGLLSDFSVPLGSFD 787
Fugu            -------------CMKQKNASRSLLILLFCPESLS------QTSSTGQIPFYSSSK---- 711
Rat             VLAQYPSLQDVPQVVLEGATIQPGENIFLDPYIFWQILNGQLSQYPGPYSDFSMPLEHFN 875
Mouse           VLAQYPSLQDVPQVVLEGATTPPGENIFLDPYIFWQILNGQLSQYPGPYSDFNMPLEHFN 875
Human           VLSQYPSLQDVPLAALEGKRPQPRENILLEPYLFWQILNGQLSQYPGSYSDFSTPLAHFD 875
Marmoset        VLSQYPSLQDVPLAALEGNRLQSRENVLLEPYLFWQILNGQLSRYPGPYSDFSTPLAHFD 875
Pig             GLARYSSLQDVPLAVLEGNLTQATRNILLEPYLFWQILNGQLPRYPGPYSDFSAPLAHLD 877
Cow             GLARYSSFQDVPVSVLEGNQTQPGGNVFLEPYLFWQILNGQLDRYPGPYSDFSAPLAHFD 875
Panda           GLARYPSFQDIPLAVMEGNMTQPGGNILLEPYLFWQILNGQLSRYPGPYSDFSAPLAHLD 874
Dog             GLARYPSFEDVPLAVMEGNRTQPGGNILLEPYLFWQILNGQLSRYPGAYSDFSAPLAHFD 874
Horse           GLAKYSSLQDVPLAVLEGNLTQPGENIFLEPYLFWQILNGQLSRYPGPYSDFSIPLAHFD 876
Opossum         ILTDYSSFQAVPIEVLDGNVTMAGDNILFDPFTFWQLLTGKLSRYPGPYTDFSSPLGHFE 872
Zebra finch     IFSMYSSFTDLPPQVLKGNLTSASENILLDPYTFWQLLTDQLSYYPGPYTDFSAPLGHFE 864
Sea urchin      ------------------------------------------------------------
Amphioxus       QGLQTYMFRSVRHTGGQGLQTYMFRSVRHTGGQGLQTYMSRSVRHTGGQGLQTYMFRSVR 851


Zebrafish       LRQCWCVDLEGDMLAGSKA----------------------------------------- 806
Fugu            -------QVTHLFLPGTNE----------------------------------------- 723
Rat             LRSCWCVDEAGQELDGTRT----------------------------------------- 894
Mouse           LRSCWCVDEAGQKLDGTQT----------------------------------------- 894
Human           LRNCWCVDEAGQELEGMRS----------------------------------------- 894
Marmoset        LRNCWCVDEAGQELEGTRT----------------------------------------- 894
Pig             LRSCWCVDEAGRKLEGTQT----------------------------------------- 896
Cow             LRSCWCVDEAGQKLEGTRN----------------------------------------- 894
Panda           LRSCWCVDEAGQELEGTRT----------------------------------------- 893
Dog             LRSCWCVSEAGRELEGTRT----------------------------------------- 893
Horse           LRSCWCVDEAGQELEGTRA----------------------------------------- 895
Opossum         LRNCWCVDENGQELKGTQV----------------------------------------- 891
Zebra finch     LRDCWCVDSKGGELEGTKAGVNQVPALLKGNLTSASENILLDPYTFWQLLTDQLSYYPGP 924
Sea urchin      ------------------------------------------------------------
Amphioxus       HRVDRGFKPTCSGQSDTRVDR--------------------------------------- 872
```

```
Zebrafish       -------------------------------PVGQIPKCPGPCSVVQRQVSEFLKQAEEL 835
Fugu            -------------------------------LLTRS------CSGALAKVTAFRNEVKSI 746
Rat             -------------------------------RAGEIPACPGPCEEVKFRVLKFIKETEEI 923
Mouse           -------------------------------KPGEIPACPGPCEEVKLRVLKFIKETEEI 923
Human           -------------------------------EPSKLPTCPGSCEEAKLRVLQFIRETEEI 923
Marmoset        -------------------------------EPSKLPTC--ELTHMKFLLDVFCVQSIFI 921
Pig             -------------------------------EPSKVPACPGSCEEVKLRVLQFIKEAEEI 925
Cow             -------------------------------EPNKVPACPGSCEEVKLRVLQFIREAEEI 923
Panda           -------------------------------EPSKVPACPGSCEKVKLRVLQFIKETEEI 922
Dog             -------------------------------EPSKVPACPGSCEEVKLRVLQFLKETEEI 922
Horse           -------------------------------EPNKVPACPGSCEEVKLHILRFIEETEEI 924
Opossum         -------------------------------ETNKVPACPGACEEVRLQVIQFIEESEEI 920
Zebra finch     YTDFSAPLGHFELRDCWCVDSKGGELEGTKAGVNQVPACPGICEGVKQEAMKFMEEAEQL 984
Sea urchin      ------------------------------------------------------------
Amphioxus       -------------------------------GFKPTCSGQSDTWMDRGFKPTCSGQSDTG 901

Zebrafish       ISASNSTHVPVGYGFLLAESVYLSPEELEQT---RSSMIPVTQTLLSNTDTALR-LAAHS 891
Fugu            LNLSNSSIHPLEYEFLLADISPLTLDEINQT---EEGQW-ITDRLLSHSRSALR-LAAFS 801
Rat             VSASNASSFPLGESFLVAKGIQLTSEELGLPPLYP-SREAFSEKFLRGSEYAIR-LAAQS 981
Mouse           VSASNASSFPLGESFLVAKGIQLTSEELDLPPQFP-SRDAFSEKFLRGGEYAIR-LAAQS 981
Human           VSASNSSRFPLGESFLVAKGIRLRNEDLGLPPLFP-PREAFAEQFLRGSDYAIR-LAAQS 981
Marmoset        LELMN------------------ADEDLGLPPLFPLPGEAFSEQFLRGSDYAIR-LAAQS 962
Pig             VMVSNSSQFPLGESFLAAKGIRLTDEELALPPLSP-SRETFLEKFLSGSDYAIR-LAAQS 983
Cow             VTYSNSSRFPLGESFLAAKGIRLTDEELAFPPLSP-SRETFLEKFLSGSDYAIR-LAAQS 981
Panda           VLASNSSWFPLGESFLAAKGIRLTDEELSLPPLSP-SRESFSEKFLSGGDYAIR-LAAQS 980
Dog             VLASNSSWFPLGESFLAAKGIWLTDEELSLPRLSP-SRETFSEKFLSGGDYALR-LAAQS 980
Horse           VLASNSSQFPLGESFLAAKGIWLTSEELALPRLSP-SWETVSEKFLSGNDYAIR-LAAQS 982
Opossum         IRASNSSQFALGESFLMAKGILLSDEELSRPLSFP-SKETFSNSMFHGSDYALR-VAAQS 978
Zebra finch     ILASNGSQFAFGESFLLAKGIELTDRDLLRSAGPDGLQAAIPQELLSGRDSALQ-LAAWS 1043
Sea urchin      ------------------------------------------------------------
Amphioxus       WTGASNLHVQVSQNTGGQGLQTYIYVQVSQTRVDRGSKPTCSGQSDTRVDRGFKPTCSGQ 961

Zebrafish       TLQFYWQSRLMASDRDRQSLMLGYQPYIPQCDAYGQWLPNQCYQSTGLCWCVDEEGQYIA 951
Fugu            -----RESAGTFVHRR------SYEPFLPQCDGDGNWLNRQCFHSTGQCWCVDEDGEYIP 850
Rat             TLTFYQKLRASLGESNGTASLLWSGPYMPQCNTIGGWEPVQCHPGTGQCWCVDGWGELIP 1041
Mouse           TLTFYQSLRASLGKSDGAASLLWSGPYMPQCNMIGGWEPVQCHAGTGQCWCVDGRGEFIP 1041
Human           TLSFYQRRRFSPDDSAGASALLRSGPYMPQCDAFGSWEPVQCHAGTGHCWCVDEKGGFIP 1041
Marmoset        TLSFYQSRRFSLDNNAGASARLRSGPYMPQCDAFGSWEPMQCYAGTGHCWCVDEKGEFIP 1022
Pig             TFSFYQRRRVALSDAPRTSGPLQPYPYVPQCDALGSWEPVQCHAATGHCWCVDGEGAYLP 1043
Cow             TFDFYQRRLVTLAESPRAPSPVWSSAYLPQCDAFGGWEPVQCHAATGHCWCVDGKGEYVP 1041
Panda           TLDFYQRRGFLLGDSTRASALPRPGPYVPQCDVWGGWEPVQCHAGTGYCWCVDRKGEYVP 1040
Dog             TLDFYQRRGFLLGDSTRTSALLRPVPYVPQCDVWGGWEPVQCHARTGYCWCVDGKGEYVP 1040
Horse           TFDFYQRRRFALGDSARTSALLPPSPYVPQCDALGRWEPVQCYARTGHCWCVDGKGEYVP 1042
Opossum         TLSFYQRSQSTLGDPVNEATILGYHPYVPQCDGFGNWEPLQCSESTGHCWCVDEKGQYVA 1038
Zebra finch     VLRFYWQSYFTSKSSAGEATQLGFFPYIPQCDGLGNWEPVQCYESTGHCWCVDERGRYIM 1103
Sea urchin      ------------------------------------------------------------
Amphioxus       SDKVEPKGKCHVDRGLAIDAGLGPGAYVPQCREDGTYDPLQCHGSTGECWCVSEYGDEIS 1021

Zebrafish       DSLTSRS-----SLPQMCEHPAKLYARG--------------SKVTLCSLTGDKQALIS- 991
Fugu            DSLTSRSDHLPRCLTQCQRAHAQLLLSGWMKSSDHSR-----SYSPQCEEDGKFSVLQTG 905
Rat             GSLMARSSQMPQCPTSCELSRANGLISAWKQAGHQRNPGPGDLFTPVCLQTGEYVRQQTS 1101
Mouse           GSLMSRSSQMPQCPTNCELSRASGLISAWKQAGPQRNPGPGDLFIPVCLQTGEYVRKQTS 1101
Human           GSLTARSLQIPQCPTTCEKSRTSGLLSSWKQARSQENPSPKDLFVPACLETGEYARLQAS 1101
Marmoset        ASLTARSLQIPQCPTTCEKSRSSGLLSSWKQARSQGNPTPKDLFIPACLETGEYARLQAS 1082
Pig             ASLAARSPQVLQCPSPCETSRVRGLLSAWKQAGSQVRPSPKDLFIPACTETGEFARLQAS 1103
Cow             TSLTARSRQIPQCPTSCERLRASGLLSSWKQAGVQAEPSPKDLFIPTCLETGEFARLQAS 1101
Panda           ASLTARSPQIPQCPTACETSRASGLLSSWKQAASQGNPSPKDLFIPACLETGEFARLQES 1100
Dog             ASLTARSPRILRCPTACEKSRASGLLSSWKQAGSQGNPSPKDLFIPTCLETGEFARRQES 1100
Horse           ASLTARSPQMPQCPTTCEKSRASGLLASWKQAGSQGNPSPEDLFIPTCLETGEFARLQAS 1102
Opossum         DSLAARSTRLPQCPSVCEKSQTNGLISNWEQGSAEGNASPGDLFTPSCLETGEYNRLQES 1098
Zebra finch     DSLVSRSSELPKCRTSCQRSQANALISSWRQSSAKLDTSAADLFIPNCLETGEYTVLQRS 1163
Sea urchin      ------------------------------------------------------------
Amphioxus       GSRVSPGQLVPNCGDLSARTKRPQSQCEQEQQEVLAFALPG------------------- 1062
```

```
Zebrafish      ------LTHTDRHVKRMKTLALQREVALG------------------YEPECVQDGQHFS 1027
Fugu           GDAGWCVNPQNGEQIQMATLSPGGQLTCPSRCQL-------------LAFQCKSDG-SFQ 951
Rat            GTGAWCVDPSSGEGVPT---NTNSSAQCPGLCDALKSRVLSRKVGLGYTPVCEALDGGFS 1158
Mouse          GTGTWCVDPASGEGMPV---NTNGSAQCPGLCDVLKSRALSRKVGLGYSPVCEALDGAFS 1158
Human          GAGTWCVDPASGEELRP---GSSSSAQCPSLCNVLKSGVLSRRVSPGYVPACRAEDGGFS 1158
Marmoset       EAGTWCVDRASGEELLP---GLNSSAQCPSLCNVLKNGVLSRRIGPGYVPTCGAEDGGFF 1139
Pig            EASTWCVDPASGEATPP---GTNSSAPCPGLCEVLQRGVPSRRASPGTTPACRAEDGGFA 1160
Cow            EAGTWCVDPASGEGVPP---GTNSSAQCPSLCEVLQSGVPSRRTSPGYSPACRAEDGGFS 1158
Panda          EAGSWCADPASGAGMPP---GMNSSAPCPSLCEVLPSGVLSRRASSGYTPACRAEDGGFS 1157
Dog            EGGTWCVDPGSGAGRPP---GTDSSAPCPSLCEELSSGTLSRRASSGYSPACRAEDGGFS 1157
Horse          EAGTWCVDPATGEGMPP---GTNSTAQCLSRCEGLQRGDPSRRASPGYTPACRAEDGGFS 1159
Opossum        ESNTWCVNLASREGTRLGELPSNSSNPCPSTCNTLMEKANLREVGSGYIPTCEGRDGRFS 1158
Zebra finch    DTDIWCVDPVSGEIFQRGSKGLDGNPECPSLCNMLKSKAVSREAARGDIPQCEGDRGSFS 1223
Sea urchin     ------------------------------------------------------------
Amphioxus      -----------------------------------------------AYVPQCRDDGSFS 1075

Zebrafish      PLQCDLS--DCWCVSDSGKELPMTRSPRSTGQTPACNIPECPLPFG--DISHGAVLCSST 1083
Fugu           PLQCDMN--SCWCVSEDGQEVSGTRTRRQLGQVPSCDSPFCPTPT----ITHGTLVCLPT 1005
Rat            PVQCDLAQGSCWCVLASGEEVPGTR---VVGTQPACESPQCPLPFSGSDVTDGVVFCETA 1215
Mouse          PVQCDLAQGSCWCVLGSGEEVPGTR---VVGTQPACESPQCPLPFSGSDVADGVIFCETA 1215
Human          PVQCDQAQGSCWCVMDSGEEVPGTR---VTGGQPACESPRCPLPFNASEVVGGTILCETI 1215
Marmoset       PVQCNQAQSSCWCVMDSGEEVPGTR---VAGSQPACESPRCPLPFNVSEVVGGTILCETI 1196
Pig            PVQCDPAQGSCWCVLGSGEEVPGTR---VAGSQPACE----------------------- 1194
Cow            PVQCDPAQGSCWCVLGSGEEVPGTR---VAGSQPACESPQCPLPFSVADVAGGAILCERA 1215
Panda          PVQCDPAQGSCWCVLDSGEEVPGTR---VAGSQLACESPQCPLPFNTSDVAGGVILCERA 1214
Dog            PVQCDLAQGSCWCVLDSGEEVPGTR---VAGSQLACESPRCPLPFNTTDVDGGVIVCERA 1214
Horse          PVQCDLDQGSCWCVLGSGEEVPGTH---VAGSQPACESPQCPLPFNVSEVAGGVILCERA 1216
Opossum        PVQCDLDQESCWCVLAMGEEVPGTR---VHGGRPSCESPQCPLPFSASDVVHGVVLCEKG 1215
Zebra finch    PVQCSQEQESCWCVFGNGEEVPGTR---VNGARPECASPQCALPFGASAVANGAVLCETI 1280
Sea urchin     ------------------------------------------------------------
Amphioxus      PVQCHGSSGHCWCADQFGNEVAGTRVRPGENPRCSAEAPVTEAP---------------- 1119

Zebrafish      NVP-GQQMQRCELYCDQSYVNTLPVASFLCDPQAKNWL-EDAPLSNACQKPQVLQTVQVS 1141
Fugu           ----ANSSQSCNLMCHRGYQNALPVSNFVCDTKSHQWDGKHKPLGGACQTSQLWS----- 1056
Rat            SSSGVTTVQQCQLFCRQGLRNVFSPGPLICNLESQRWV-TLPL-PRACQRPQLWQTMQTQ 1273
Mouse          SSSGVTTVQQCQLLCRQGLRSAFSPGPLICSLESQHWV-TLPP-PRACQRPQLWQTMQTQ 1273
Human          SGPTGSAMQQCQLLCRQGSWSVFPPGPLICSLESGRWE-SQLPQPRACQRPQLWQTIQTQ 1274
Marmoset       SGPTGAAIQQCRLLCRQG-----------------------------FRRPQLWQTIQTQ 1227
Pig            -------------------------------------------------RPQLWQTIQTR 1205
Cow            SGLGAAAGQRCQLRCSQGYRSAFPPEPLLCSVQRRRWE-SRPPQPRACQRPQFWQTLQTQ 1274
Panda          SGAGGAPVQRCQLWCRRGYRSAFPPGPLVCSLEKRRWE-SQPPQPQACQRPQLWQTLQAR 1273
Dog            SGPGGAPVQRCQLLCRRGYRSAFLPGPLVCSLEEGRWL-SQPPQPQACQRPHLWQTFQTQ 1273
Horse          SG--EATVXRCQLLCRWGYRSAFPPGPLVCSPQRRLWV-SQPPQPRACQRPQLWQTLQTG 1273
Opossum        TDK-RQAVQQCQLICRQGYHLALPRGPFACNIESQRWI-SEPPLPQACQKLQLLQTVQTQ 1273
Zebra finch    SG-QAPGIQQCQLVCRQGFHSAAPSSPSQCDARQRRWV-SAAPLPQACQKLQLFQTVQAQ 1338
Sea urchin     ------------------------------------------------------------
Amphioxus      ------PQTKCQEEREDANSRGLLAGVYVPQCTEEGLYNPIQCHGSTGQCWCVNDSGEEI 1173

Zebrafish      VQLQLSLNEGQQSCSTQS---VDLQAALLRDMRATGLCSLQLTSSGQTSSVSVCDESSVS 1198
Fugu           ----------LPSACSHI---SSLKSTFLHLMTSRGLCSAQLPASG--RSVSLCDASSVH 1101
Rat            AHFQLLLPPGKMCSIDYSGLLQAFQVFILDELITRGFCQIQVKTFGTLVSRTVCDNSSIQ 1333
Mouse          AHFQLLLPPGKMCSVDYSGLLQAFQVFILDELIARGFCQIQVKTFGTLVSSTVCDNSSIQ 1333
Human          GHFQLQLPPGKMCSADYADLLQTFQVFILDELTARGFCQIQVKTFGTLVSIPVCNNSSVQ 1334
Marmoset       GHFQLQLPPGKMCSADYAGLLQAFQVFILDELTARGFCQIQVKTFGTPVSIPVCDNSSVQ 1287
Pig            GQFQLQLPPGKVCSADYAGLLPTFQVVILDELTARGFCRIQVTTARTPVSIPVCDDSTVR 1265
Cow            AQFQLLLPLGKVCSADYSGLLLAFQVFLLDELTARGFCQIQVKTAGTPVSIPVCDDSSVK 1334
Panda          GQFQLRLPPGKMCSADYAGLLLVFQVFILDELMARGFCEIQAKTFGTPVSIPVCDGSTVQ 1333
Dog            GQFQLQLPPGKMCSADYAGLLPAFQVFLADELEARGFCQIQAKTLGTPISIPVCDSSTVQ 1333
Horse          AQIQLQLPPGSLCRADDAGLLPVFQGFILDELMARGFCQIQVKTYGTPVSIPVCNDSTVR 1333
Opossum        VQFHLQLPPGKMCSSDYSGLLQAFQSFILDELKARGFCQIQGNTFGAVFSFPVCDDSTVQ 1333
Zebra finch    THFQLRLPPEKTCSSDYSGLLQAFQIFILDELKARGLCHLQVNAFGKTGSVSMCDDSTVY 1398
Sea urchin     ------------------------------------------------------------
Amphioxus      MGTRVKAGESPNCAGTPATKCQLEREEMLSEGPLLGAYIPTCAEDGSYDVIQCHDSTGHC 1233
```

```
Zebrafish       LECSN--DKEVTARITLMARLSDLPLAALPDLHDIDTALSEERLLNGLKKLILSGSYQSI 1256
Fugu            LQCDNG-ITDLMLMVTWSAKVSDLATSDLPNLQDFSQFLNDSSLLRGIRDIMGN--IQSM 1158
Rat             VGCLT--AERLGVNATWKLQLEDISVGSLPNLHSIERALMGQDLLGRFANLIQSGKFQLH 1391
Mouse           VGCLT--AERLGVNVTWKLQLEDISVGSLPDLYSIERAVTGQDLLGRFADLIQSGRFQLH 1391
Human           VGCLT--RERLGVNVTWKSRLEDIPVASLPDLHDIERALVGKDLLGRFTDLIQSGSFQLH 1392
Marmoset        VGCLT--RERLGVNVTWKSRLEDIPVASLSDLYDIERAMVGKDLLGRFTDLIQSGSFQLH 1345
Pig             VGCLS--LDRLGVNVTWTLRLEDAPPASLPDLRDIEEALAGKDLVGRFADLIQSGTFQLH 1323
Cow             VECLS--RERLGVNITWKLQLVDAPPASLPDLQDVEEALAGKYLAGRFADLIQSGTFQLH 1392
Panda           VECLA--GERLGVNVTWKLPLKDVPPASLPDLHDIEEALVGKDLIGRFTDMIQSGGFQLH 1391
Dog             VECVT--GERLGVNVTWKLHLEDVPPASLPDLHDIEEALVGKDLIGRFTDLIQSGGFQLH 1391
Horse           VECLT--QERVGVNVTWKLRLEDVPPASLPDLRDVEEALVGKNLIRRFADLIQSGAFRLH 1391
Opossum         VECLT--GERLGVNVTWSSQLEDIPATSFPDFHDIEKAFMGNDLVGRFAKLIQSGGFQLY 1391
Zebra finch     VQCLG--VDRLGVNITWRTQLENIPTASLPDLHDIENAIIGDNLIGAFIKEIKDGVFLLH 1456
Sea urchin      ------------------------------------------------------------
Amphioxus       WCVDREGEEILGTRVGPQEDVVCEDDATTSKPGVKEGKMPLGSETEEVTEAPKP------ 1287


Zebrafish       FLSD-------------HSLALSSPPSFSCMTGYSQLPQ-------STGCVLCPAGSFFS 1296
Fugu            LASK-------------PKLVSVKSPSLGCSRGYRLDLS-------GAGCLICPAGTLST 1198
Rat             LDSKTFSADTILYFLNGDRFVTSPMTQLGCLEGFYRVST---TSQDPLGCVKCPEGSFSQ 1448
Mouse           LDSKTFSADTTLYFLNGDSFVTSPRTQLGCMEGFYRVPT---TRQDALGCVKCPEGSFSQ 1448
Human           LDSKTFPAET-IRFLQGDHFGTSPRTWFGCSEGFYQVLT-SEASQDGLGCVKCPEGSYSQ 1450
Marmoset        LDSKSFPADTAIRFLQGDHFGTSPRTRFGCLEGFYQVWT-SEASQDRLGCVKCPEGSYSQ 1404
Pig             LDSRTFPADPSIHFLQGDSLGTSPRTRFGCVEGSRQVPATSNTSQDPLGCVRCPEGSYFQ 1383
Cow             LDSKTFSADTSIRFLQGDRFGTSPRTQFGCLEGFGRVVAASDASQDALGCVKCPEGSYFQ 1452
Panda           LDSKTFPADTSIYFVQGDRLGTSPRTWFGCLEGFRQVLATSNATQDPLGCVMCPEGSYFQ 1451
Dog             LDSKTFPADTSIYFLQGDRFGTSPRAWFGCLEGFHQVLAPSNAPQDPWGCVKCPEGSYFQ 1451
Horse           LDSKTFPVDTSIRFIQGASFGTSPRTWFGCLEGFHQVSATSSAGQDPLGCVQCPEGSYFQ 1451
Opossum         LDSKIFPADTTIRFLRGGDFDTSPAIQFGCKKGFQKNSPQGKTVLDLPGCVICPEGSYFQ 1451
Zebra finch     LDSKQFIADSVIDFPRDEEFDLSPRVQLG---------------------FVCPPGSYFQ 1495
Sea urchin      ------------------------------------------------------------
Amphioxus       ---------------------------------------------------TVTVRSVTV 1296


Zebrafish       GGVCTPCPRDTFQEDEGQVFCSPCPAGTSTVAQGAFSASHCLTECKKS--KLSCTTKGDF 1354
Fugu            EGGCALCPQGTYQDQEGRDFCHKCPRGSS--LTGASSVNQCVTDCQSR--GLRCSEKGDF 1254
Rat             DGKCTPCPAGTYQGQAGSSACIPCPRGRTTTITGAFSKTHCVTDCQRDEAGLQCDQNGQY 1508
Mouse           DGRCTPCPAGTYQEQAGSSACIPCPRGRTTIITTGAFSKTHCVTDCQKNEAGLQCDQNGQY 1508
Human           DEECIPCPVGFYQEQAGSLACVPCPVGRTTISAGAFSQTHCVTDCQRNEAGLQCDQNGQY 1510
Marmoset        DEQCILCPVGFYQEQAGSLACVPCPMGRTTISAGAFSQTHCVTDCQRNEAGLQCDQNGQY 1464
Pig             EEQCIPCPAGFYQEQTGSLACAPCPAGTTTTSVGAFSQTHCVTACQRDEAGLQCDQDGQY 1443
Cow             DEQCIPCPAGFYQEQAGSLACVPCPEGRTTVYAGAFSQTHCVTDCQKNEVGLQCDQDSQY 1512
Panda           KETCIPCPVGFYQERAGSVACIPCPMGRTTISPGAFSPMHCVTACQRSEVGLQCDQDGQY 1511
Dog             KEICIPCPVGFYQEQAGSMDCVPCPVGRTTISPGAFSHTHCVTDCQRSEVGLRCDQDGQY 1511
Horse           DERCIPCPVGFYQERAGGLACDPCPLGRTTLSAGAFSQTHCVTDCQKNETGLQCDQDGQY 1511
Opossum         NEECVPCSPGFYQEQPGSLACVRCPAGRTTISIGAFSQSHCVTDCQKNDLGLQCDDDGAY 1511
Zebra finch     DDECIPCPLGYYQYQTGHSFCIKCPVGKTTNSYGAISADHCVTPCQGGEQGLQCDGEGQY 1555
Sea urchin      ------------------------------------------------------------
Amphioxus       KAETPKNETEKFLPPENGLNAFLNLTCRDARRRALRLFWYNFTTADEVSYIPTCTADGKF 1356


Zebrafish       LSAQKDPVSGKWRCVSAQGEELTWTSSDGLLTVEECKVMEKFEVVSSSSLLLKSESADTL 1414
Fugu            LPAQPDFLSGRWRCVSSEGLDLDWSNSEKALTDEECSVFRRFQAVSGSDVIVNADDAEVL 1314
Rat             QANQKDMDSGEVFCVDSEGQRLQWLQTEAGLSESQCLMMRKFEKAPESKVIFDASSPVIV 1568
Mouse           QASQKNRDSGEVFCVDSEGRKLQWLQTEAGLSESQCLMIRKFDKAPESKVIFDANSPVIV 1568
Human           RASQKDRGSGKAFCVDGEGRRLPWWETEAPLEDSQCLMMQKFEKVPESKVIFDANAPVAV 1570
Marmoset        RASQRDKGSGKAFCVDSEGQRLPG-ETEAPLEDSQCLMMQKFEKTPESKVVFDASAPVAV 1523
Pig             RASQRDRASGKAFCVDSEGRRLPWSETQAPLVDAQCLMMRKFEKLPESKVIFTADVAVLG 1503
Cow             RASQRDRTSGKAFCVDGEGRRLPWTEAEAPLVDAQCLVMRKFEKLPESKVIFSADVAVMV 1572
Panda           RASQRDRDSRKSFCVDGEGRRLPWSDTDGPLSDSQCLMMRKFEKALESQVIFGANVTAVG 1571
Dog             RASQRDGAGGKAFCVDGEGRRLPWSETEAPLTDSQCQMMQKFEKAPDSKVVFSANVTAVG 1571
Horse           RASQRDRGSGKAFCVDGEGRRLPWSETEAPLTDSLCLMMQKFEKVPESQVLFNADVAVVV 1571
Opossum         SPSQRDPSGRGAFCVDSLGKTLAWTETDGSLTSSQCLMLRKFEQAPESKVIFSASDALVV 1571
Zebra finch     RPAQQDPATQKSLCVDSFGAALDWTETEDLLTDEQCLVLRKFERAPASSLIYGSEEAQIL 1615
Sea urchin      ------------------------------------------------------------
Amphioxus       FPMQCYGASGYCFCVDEAGKEIPGTVSEP----------------GARPVCSGVDDAKVD 1400
```

```
Zebrafish      SSDSSSQPKETQLRKCVLDCTQDDSCLYVAVFSDEEKTHCEMYSTSADNVECRTTEPSKG 1474
Fugu           RTLT------ADLSTCLHACSAEPSCHHVALFN--LHSQCELYSTHTVNTHCNTSQQTRG 1366
Rat            KSRVPS--ANSPLVQCLADCADDEACSFVTVSSMSSEVSCDLYSWTRDNFACVTSDQEED 1626
Mouse          KSSVPS--ADSPLVQCLTDCANDEACSFLTVSTMESEVSCDFYSWTRDNFACVTSDQEQD 1626
Human          RSKVPD--SEFPVMQCLTDCTEDEACSFFTVSTTEPEISCDFYAWTSDNVACMTSDQKRD 1628
Marmoset       RSKVPD--SEFPVMQCLTDCAEDETCGFLTMSMMEPEVSCDFYAWTSDNVACMTSEQKQD 1581
Pig            -SKVPD--SESSLMQCLADCALDEACGFLTVSLEGSEGSCDFYAWTSDNIACTSSGQEED 1560
Cow            RSEVPG--SESSLMQCLADCALDEACGFLTVSTAGSEVSCDFYAWASDSIACTTSGRSED 1630
Panda          RSKAPG--SESPLMQCLTDCALDAACSSLTVSSVGSEVSCDFYAWSSDNIACVTSTQDQD 1629
Dog            RSKVSG--SGSPLLQCLTDCALDETCSFLAVSTAGSEVSCDFYGWTSDSIACTTSAQHQD 1629
Horse          RSRVPD--SDSPLIQCLADCALDEACSFLTVSTAGSEVSCDFYAWTSDNIACMTSGQDRE 1629
Opossum        QSKTMK--GEFPLMQCLLDCSLDEACSFLTVTVAGSDAVCDFYAGTDVNIDCIASNQEQG 1629
Zebra finch    QSQSFEGDLQSTYFQCISGCEREDACGFAAVTPAGTEVLCELYSAAEANFNCTTSGSVQG 1675
Sea urchin     ------------------------------------------------------------
Amphioxus      TWDTGS-----------GEDGGEEPTEEIELFTSAPTPDKAVLPTESPMVETTTIATSVL 1449

Zebrafish      FLGNDGAEAFQTLICVLKIKGDEPD-LTVLRKKGYEFSTAGLKR--FERLNFRKAGSGVY 1531
Fugu           FLGNLQAEHSDWLSCFPRVKGRASD-LLVIKKKGVEFTSSWQQQQMYLKMKMMKVLSGVF 1425
Rat            AVDSLKETSFGSLRCQVKVRNSGKDSLAVYVKKGHEFTASGQKS--FEPTGFQNVLSGLY 1684
Mouse          AMGSLKATSFGSLRCQVKVRNSGKDSLAVYVKKGYESTAAGQKS--FEPTGFQNVLSGLY 1684
Human          ALGNSKATSFGSLRCQVKVRSHGQDSPAVYLKKGQGSTTTLQKR--FEPTGFQNMLSGLY 1686
Marmoset       ALGNSKATSFGSLRCQVKVRSRGQDSPAVYLKKGQGYTTTLQKS--FEPTGFQNTLSGLY 1639
Pig            ALGTSKATSLRSLTCQVKVRPGDGVPPAVYLKKGQEFATVGRKR--FEQTGFQNALSGLY 1618
Cow            ALGTSQATSFGSLQCQVKVRSREGDPLAVYLKKGQEFTITGQKR--FEQTGFQSALSGMY 1688
Panda          TLGNSKATGFGSLRCQVTVRSGAQDPPSVYLKKGAGSTWRLQKS--FEQTGFQNTLSGMY 1687
Dog            TLGNSEATSFGSLRCQMTVRSGAQDSLAVYLKKGQEFTTTSQKS--FEQTGFQNMLSGLY 1687
Horse          ALGNSEATGFGSLRCQVQVRSGDGGSLAVYLKKGQEFPTASQKS--FQNTGFQNTLSGVY 1687
Opossum        ALGNAAATSFQHLNCLLKVKNDDKASVTVYLKKGYEFTTSGQKT--FEKTNFQNTLSGVY 1687
Zebra finch    VLGNPAATSISSLSCLLHVRNPEGDALMAFLKRGLEFLSSGPKA--FERTGFQNVLSGVF 1733
Sea urchin     ------------------------------------------------------------
Amphioxus      GLVTTAASKLGLVTTAASKPGLFTTVTPVLNTTAELGPDVSTRMPTLCERARAEALTALK 1509

Zebrafish      RTLVFDARGSALADVHRFCVDSCGRETCCDGFILNQNVLNGGSIMCG-FLTAPTVLQCSE 1590
Fugu           RTQVFSSRQTSLSDVHRFCQDTCHHDKCCHGYIINQNSLMSGSLFCG-WLRSPSVLMCEE 1484
Rat            SSVVFSALGTNLTDTHLFCLLACDQDSCCDGFIVTQVKE--GPTICG-LLSAPDILVCHI 1741
Mouse          SPVVFSASGANLTDTHTYCLLACDNDSCCDGFIITQVKG--GPTICG-LLSSPDILLCHI 1741
Human          NPIVFSASGANLTDAHLFCLLACDRDLCCDGFVLTQVQG--GAIICG-LLSSPSVLLCNV 1743
Marmoset       NPVVISR--ASLTDAHLFCLLACDHDLCCDGFILTQVQG--GAIICG-LLSSPSVLLCNV 1694
Pig            SPVVFSASGASLTEAHLFCFLACDRDSCCDGFILTQVQG--GPIICG-LLSSPDVLLCHV 1675
Cow            SPVTFSASGASLAEVHLFCLLACDHDSCCDGFILVQVQG--GPLLCG-LLSSPDVLLCHV 1745
Panda          GPVVFPASGADLTGAHLFCLLACDHDSCCDGFILAQVQG--GPIICG-LLSSPDVLLCNV 1744
Dog            RPVIFPASGADLTAAHLFCLLACDRDSCCDGFILAQLQG--GPVICG-LLSSPDVLLCHV 1744
Horse          NPVVFSASGANLTEAHLFCLLACDRDSCCDGFILAQVQG--GPIICG-LLSSPDVLLCNV 1744
Opossum        RSIMFSIDGANLMDTHLYCHLACDGEPCCDGFILTQTLL--GGVLDSYLISSDTFLISKP 1745
Zebra finch    RSLALPAAGTSLTDAGLLCRQECSQDSCCDGFILSQLLLDAGTILCS-LLSSPDVLICNA 1792
Sea urchin     ------------------------------------------------------------
Amphioxus      ASEQHNRSRSAFVPTCTEEGRYTPRQCYLDGVNCFCVNKYGNALSAVTRMTEGEELECEE 1569

Zebrafish      SDWDVKSLSSSS--------------------RICGAGVQYSKQLKRFTFSFGGQNFTIT 1630
Fugu           QDWDVTGQGTAN--------------------RICGAGLQYNEQQKSFVFDFGGQMFTIN 1524
Rat            NDWRDASDTQAN---------------------GTCAGVTYDQGSRQMTMSLGGQEFLQG 1780
Mouse          NDWRDTSATQAN---------------------ATCAGVTYDQGSRQMTLSLGGQEFLQG 1780
Human          KDWMDPSEAWAN---------------------ATCPGVTYDQESHQVILRLGDQEFIKS 1782
Marmoset       EDWMDASEARAN---------------------ATCPGVTYDQETRQVTLRVGGQEFIKS 1733
Pig            RDWRDPSEAQAD---------------------ATCPGMTYDQDSHQGTLRLGGQEFK-S 1713
Cow            RDWRDPAEAQAN---------------------ASCPGVTYDQDSRQVTLRLGGQEIR-G 1783
Panda          KDWRDPAEAQAN---------------------ATCPGVTYDQGSRQVTLRLGGQEFQSN 1783
Dog            KDWRDPTEAQAN---------------------ATCPGVTYDRGSRQATLRLGGQEFR-V 1782
Horse          KDWRDPSEAQTS---------------------ATCPGVTYDHGSRQVTLRLGGQEFK-S 1782
Opossum        LSWKEFQCPNCNP-------------------NAACEPPHYPRQGREEELPLGYLEVR-S 1785
Zebra finch    KGWSPAPASAMG---------------------EMCKGVSYDEKEKRFSFTLGGQVFSGT 1831
Sea urchin     ------------------------------------------------------------
Amphioxus      HDYQTAAPPIAEP----------------------------------------------- 1582
```

```
Zebrafish       DAALPASSKNKTGYQETLISFQRVYLWKESDMNTRAKTLSACSGSAVGEDSRSALSDSVK 1690
Fugu            -TALSTDTKTKKDYQASIISFQAVFLNPDADEGRPASSCSGTADLSPPLDG-WGDSASLK 1582
Rat             LTLLEGT-------QDSFISFQQVYLWKDSDIGSRPESMGCGRGMVP-KSEAPEGADMAT 1832
Mouse           LALLEGT-------QDSFTSFQQVYLWKDSDMGSRPESMGCERGMVP-RSDFPG--DMAT 1830
Human           LTPLEGT-------QDTFTNFQQVYLWKDSDMGSRPESMGCRKDTVP-RPASPTEAGLTT 1834
Marmoset        LTPLEGT-------QGTFTNFQQVYLWKDSDMGSRPESVGCRKGTVP-SPTSPTETGLTT 1785
Pig             LTPREGA-------RDTFTSFQQVYLWKDSDMGSRSESMGCRRDMQP-RPESPEETDLTA 1765
Cow             LTPLEGT-------QDTLTSFQQVYLWKDSDMGSRSESMGCRRDTEP-RPASPSETDLTT 1835
Panda           LRPMEGT-------LDTITSFQQVYLWKDSDMVSRSESLGCRRDTEP-RAASPTETDLVT 1835
Dog             PVALEGT-------SGTVTSFQRVYLWKESDMGSRSESMGCRRDMEP-RPASPTETDLTT 1834
Horse           LTPLEGP-------QGIFTSFQQVYLWKDSDMGSRSESMGCSGDREP-RPASPTETDVMT 1834
Opossum         SELFEGI-------EGPFISFQEVYLWKDSDVATRTKSTECSLDAVQ-GQGPPPIPDSTM 1837
Zebra finch     SQLMEET-------ERNFTTFQEIYLRRDSDMVTRMKTSLCEAAALK-TKDDLVLSDSTK 1883
Sea urchin      ------------------------------------------------------------
Amphioxus       ------------------------------------------RTEPSRPVGCCPRGSSL 1599

Zebrafish       EAFDVLDSGDVNVDPERELPNQLYWIFKHQYSFQEAQLWCLKRCEEEE-LCHVSDIRDEG 1749
Fugu            QRFQLVSENDVIVNPNRKLPTLSFWLNKKHFDSQHALLWCLSRCDEEP-QCSVADLRDAD 1641
Rat             ELFSPVDITQVIVNTSHSLPSQQYWLSTHLFSAEQANLWCLSRCAQEPVFCQLADIM-ES 1891
Mouse           ELFSPVDITQVIVNTSHSLPSQQYWLFTHLFSAEQANLWCLSRCAQEPIFCQLADIT-KS 1889
Human           ELFSPVDLNQVIVNGNQSLSSQKHWLFKHLFSAQQANLWCLSRCVQEHSFCQLAEIT-ES 1893
Marmoset        ELFSAVDLDQVIVNENRSLPIQKHWLFKHLFSAQEANLWCLSRCVQEHSFCQLAEIITDN 1845
Pig             ELFSPVDLNQVIVSENRSLPSQQHRLFKHLFSLQQAHLWCLSRCVQEPSFCQLAEIT-DS 1824
Cow             GLFSPVDLIQVIVDGNVSLPSQQHWLFKHLFSLQQANLWCLSRCAGEPSFCQLAEVT-DS 1894
Panda           ELFSPVDLNQIIVNRSRSLPSQQHWLFKHLFSPQQANLWCLSRCVQEPSFCQLVEIT-DS 1894
Dog             ELFSPVDLDQVIVNGSQSLPSQQHWLFKHLFSPQQANLWCLSRCVQEPSFCQLVEIT-DS 1893
Horse           ELFSPVDLSQVVVNGSQSLPSQQHWLFKHLFSPQQANLWCLSRCFQEPSFCRLAEIT-DS 1893
Opossum         ELFSQVNTERVVIDGNRTVPSQQHWLFKLQFSAQQAKLWCLSPCAQENTFCQLQEVQ-TQ 1896
Zebra finch     DLFYLMDNSQIQSDQNYSLPYQQYWVFRQKYSAEEALLWCLTRCAQEEEFCRMADLQ-SA 1942
Sea urchin      ------------------------------------------------------------
Amphioxus       VLSPPVVVKDVGRGGWYSSWARDPLDPNPIYIFDGPRT---------------------- 1637

Zebrafish       -PLYFACVLYPDTRVCGAYDKPLRQTCSLVMTQSLQTAYQKKVSLTGSVKSFYSRVPFKK 1808
Fugu            SAQFHSCSLYPDSRECGGYENPRRQSCALVLETPPDNTYSKTVNLSGPIKSFFERIPFQK 1701
Rat             SSLYFTCSLYPEAQVCDNDVESNAKNCSQILPRQPTALFQRKVVLNDRVKNFYTRLPFQK 1951
Mouse           SSLYFTCFLYPEAQVCDNVMESNAKNCSQILPHQPTALFRRKVVLNDRVKNFYTRLPFQK 1949
Human           ASLYFTCTLYPEAQVCDDIMESNAQGCRLILPQMPKALFRKKVILEDKVKNFYTRLPFQK 1953
Marmoset        GSMYFTCTLYPEAQVCDDIMESSPQGCRLILPQRPMALFRKKVILEDKVKNFYTRLPFQK 1905
Pig             SPLYLTCTLYPEAQVCDDVMEASPRGCRRILPRRPNALFQRRVVLQDRVKNFYTRLPFQK 1884
Cow             EPLYFTCTLYPEAQVCDDILESSPKGCRLILPRRPSALYRKKVVLQDRVKNFYNRLPFQK 1954
Panda           APLYFTCALYPEAQVCDDVMESNPKGCRLILPHRPKTLFRKKVILRDKVKHFYTRLPFQT 1954
Dog             APLYFTCALYPEARVCDSVMESSPKGCRLILPHRPETLFRKKVILRDKVKNFYTRLPFQK 1953
Horse           APLHFTCILYPEAQVCDDVLESTPKGCRLTLPHRPGALFRKKVVLKDKVKNFYTRLPFQK 1953
Opossum         SSGDFTCTLFQKAQTCDNSLTFTPESCMFVLPYRPQTLFHKKVILGDKVTNFYIRLPFQE 1956
Zebra finch     TDKYFVCTLYPEAQICDSNISQIPGNCATVLPWEPQTLYHKIVTLKSSVKSFYTRVPFQK 2002
Sea urchin      ------------------------------------------------------------
Amphioxus       --------------IQKYDNDYAIVNDQVSRTWTLPSDIAGNGQLVYGGSAYYNRAGTQD 1683

Zebrafish       MVSYSVRSRVSVS-SKPITEGFFECERRCDEDPCCRGIGYVKDS-GVAGSDVLCLTLNSL 1866
Fugu            MVSYSVRSRTRVTGNTSLSEGFIHCERRCDEDPCCRGFGFIRDNKSMSNHDVLCLSLISF 1761
Rat             LSGISIRDRIPMS-EKLISNGFFECERLCDRDPCCTGFGFLNVS-QMQGGEMTCLTLNSM 2009
Mouse           LTGISIRDKVPMS-GKLISNGFFECERLCDRDPCCTGFGFLNVS-QLQGGEVTCLTLNSM 2007
Human           LMGISIRNKVPMS-EKSISNGFFECERRCDADPCCTGFGFLNVS-QLKGGEVTCLTLNSL 2011
Marmoset        LMGLSIRNKVPMS-EKSISNGFFECERLCDMDPCCTGFGFLNVS-QLKGGEVTCLTLNSL 1963
Pig             LTGLSIRHKVPMA-DKAISSGFFECERLCDVDPCCTGFGFLNVS-QLKGGEVTCLTLNSL 1942
Cow             LTGISIRNKVPMS-DKSISSGFFECERLCDMDPCCTGFGFLNVS-QLKGGEVTCLTLNSL 2012
Panda           LTGISIRNKVPMS-EKSISSGFFECERLCDADPCCTGFGFLNVS-QLKGGEVTCLTLNSL 2012
Dog             LMGISIRNKMPMS-EKSISNGFFECERLCDADPCCTGFGFLNVS-QLTGGEVTCLTLNSL 2011
Horse           LTGTSIRSKVPMS-DKSMSDGFFECERLCDVDPCCTGFGFLNVS-QLKGGEVTCLTLNSL 2011
Opossum         LRGISVRSKISMT-RKPLSNGFFECERRCDADLCCKGFGFLNVS-QREGGELMCLTLSHL 2014
Zebra finch     VTEISVRNKTDMS-RKAVSDGFFECERWCDADPCCTGFGFFNDS-QLSGGKIVCVTLNSL 2060
Sea urchin      ------------------------------------------------------------
Amphioxus       LIKFDLENERVAARQQLPFAGIQNSYSYSWGGSTDIDFAVDEYG-----LYVIYGTLQNR 1738
```

```
Zebrafish      GIQTCGENDSTSWRIQDCSTSQVQTQLYPFGWYEKPVNQWPKNPDVCPPFSLRSPSKNAD 1926
Fugu           GVQTCSEEDSSSWRTQDCSTS-VQTGPEPLGWYQKPVNQWSSAPALCPEFTLKTPANMGD 1820
Rat            GIQTCSEENGATWRILDCGSEDTEVHTYPFGWYQKPAVWS-DAPSFCPSAALQSLTEEKV 2068
Mouse          GIQTCNEESGATWRILDCGSEDTEVHTYPFGWYQKPAVWS-DTPSFCPSAALQSLTEEKV 2066
Human          GIQMCSEENGGAWRILDCGSPDIEVHTYPFGWYQKPIAQN-NAPSFCPLVVLPSLT-EKV 2069
Marmoset       GLQMCSEENGGAWRILDCGSPDIEVHTYPFGWYQKPIALN-NAPSFCPSVVLPSLT-EKV 2021
Pig            GLQTCSEENGGSWRLLACGSPDTEVRTYPFGWYQKPAVQN-DAPSFCPSAALPPVP-EKV 2000
Cow            GLQTCSEEYGGVWRILDCGSPDTEVRTYPFGWYQKPVSPS-DAPSFCPSVALPALT-ENV 2070
Panda          GLQTCTEENRGAWRILDCGSPDTEVHTYPFGWYRKPVAQN-DAPSFCPSVVLPSLP-EKV 2070
Dog            GLQTCSEENMGAWRILDCGSPDTEVRTYPFGWYQKPAARN-DAPSFCPVVTPPSLP-EKV 2069
Horse          GIQTCSEENGGTWRILDCGSPDTEVRTYPFGWYRKPVSEN-NAPSFCPAVVLPPLE-EKA 2069
Opossum        GFQTCSEEATGTWRILDCSSPDVEVRTYPFGWYQKPVVQA-DASSMCPSSLLPSPL-EKV 2072
Zebra finch    GIQTCAEETRSSWQISDCSSPGAEVKIHPFGWYQKPADLKSTIPNLCPPVNVPFRP-ESE 2119
Sea urchin     ------------------------------------------------------------
Amphioxus      GNMVVSQINPEDLSIINTWNAGYFKRAAGNAFMRCGVLYATDAHDFTAKISYSFDTRTEQ 1798

Zebrafish      LQ-KWKKLDAVSVYVDASVSAFDIVHISKDIAEDLDKVKNWCLSACEDSESCSAVSIDSR 1985
Fugu           TQDQWQVLSNSMVLVDRSLSTYDVIHISRDIATNPDETRDFCLLACQKEESCVAVTLMQV 1880
Rat            ALDSWQTLALSSVIIDPSIKHFDVAHISISATRNFSLAQDFCLQECSRHQDCLVTTLQIQ 2128
Mouse          TSDSWQTLALSSVIVDPSIKHFDVAHISTAATSNFSMAQDFCLQQCSRHQDCLVTTLQIQ 2126
Human          SLDSWQSLALSSVVVDPSIRHFDVAHVSTAATSNFSAVRDLCLSECSQHEACLITTLQTQ 2129
Marmoset       SLDSWQSLALSSVVVDPSIRNFDVAHVSTAAASNFSAVRDLCLSECSQHEACLTTTLQTQ 2081
Pig            ALDSWQPLPPSSVVVDPSIRNFDVAHISTAAVGDFSAARERCLLECSRHQACLVTTLQTR 2060
Cow            ALDSWQSLALSSVIVDPSIRNFDVAHISTAAVGNFSAARDRCLWECSRHQDCLVTTLQTQ 2130
Panda          ALGSWQSLAPSAVVVDPSIRNFDVAHISTATTSDFSDARNFCLLECSRHQACLVITLQMR 2130
Dog            TLGSWQSLAPSAVVIDSSIRNFDVAHVSTATTNDFSDARDFCLLECSRHQACLVTTLRAQ 2129
Horse          ALDAWQSLAPSSVVLDPSIRNFDVAQISTAATSDFSVARDFCLLECSRHKACLVTTLQTQ 2129
Opossum        SLDSWQSLDISSALIDPSIMNFDIAHISTDASNNFSAARDLCLSECSQKPACHITTLQIQ 2132
Zebra finch    YMDAWQPLNVSSVLMDSSISHFEVVHVSRDISNDFSLARDFCLSACSKNQSCAVVTLEIQ 2179
Sea urchin     ------------------------------------------------------------
Amphioxus      EHDVLVQLRDPYRYNTMLDYNPADKKLYLWDETYLITYDVMCEKDESASEVTPAATTPPT 1858

Zebrafish      ESAVRCVMYPDTHTC------LPTTSGRRCLLVTKEPAQSVYIRTG---VQLEFT----- 2031
Fugu           ASATRCILYPDTTICGLSSTRLSSNPTSSCRLLIREPASHVYLRTGDLEPSRLVT----- 1935
Rat            QGVVRCVFYPDIQSC------EHSLRSKTCWLLLHEEAAYIYRKS--GAPLHQSDGISTP 2180
Mouse          PGVVRCVFYPDIQNC------IHSLRSHTCWLLLHEEATYIYRKS--GIPLVQSDVTSTP 2178
Human          PGAVRCMFYADTQSC------THSLQGQNCRLLLREEATHIYRKP--GISLLSYE-ASVP 2180
Marmoset       PGAMRCMFYPDTQSC------THSLQGQYCHLLLREEATHIYRKQ--GISLLSYE-ASVP 2132
Pig            PGAVRCMFYADTQSC------THSLQAQNCQLLLREEATHIYRKP--DIPLPGLG-SSAP 2111
Cow            PGAVRCMFYADTQSC------THSLQAQNCRLLLHEEATYIYRKP--NIPLPGFG-TSSP 2181
Panda          PGAVRCVFYADTQIC------THSLQAQYCQLLLREEATYIYRKL--NIPLLSSG-TPAP 2181
Dog            PGAVRCVFYADAQIC------THSLQAQNCQLLLREEATHIYRKL--NIPLLSFG-TSVP 2180
Horse          PGAVRCVFYPDTQSC------THSLQAQNCQLLLREEATYIYRKP--NLPLLGSG-TSAP 2180
Opossum        PSTVRCVFYPDTQSC------AHSLQGHSCRLLLREEATYIYRKK--RGVSMRSSSTDPV 2184
Zebra finch    PSAIRCIFYPDTQMC------AHGLQGHSCWVLLKEPATYIYRRQGKNLLLPISESDSTP 2233
Sea urchin     ------------------------------------------------------------
Amphioxus      PTTTPIPPAPTPPPT--------------------------------------------- 1873

Zebrafish      SVSIPDHGTLLGESEVKPITGSDSKRVTYFLGVPYARPPIGDLRFSPPQPADWTGTWNAT 2091
Fugu           SISIPGHGTLQG-AVTETGLGSVRKSVVQFLGVPYARPPIGSLRFEAAQLADWTGTWDAT 1994
Rat            SVHIDSFGQLQG-GSQVVKVGTAWKQVYQFLGVPYAAPPLAENRFQAPEVLNWTGSWDAT 2239
Mouse          SVRIDSFGQLQG-GSQVIKVGTAWKQVYRFLGVPYAAPPLADNRFRAPEVLNWTGSWDAT 2237
Human          SVPISTHGRLLG-RSQAIQVGTSWKQVDQFLGVPYAAPPLAERRFQAPEPLNWTGSWDAS 2239
Marmoset       SVPIATHGKLLG-RSQAIQVGTSWKQVDQFLGVPYAAPPLAERRFRAPEPLNWTGSWDAS 2191
Pig            TVTIATHGQLLG-TSQAIQLGASWKQVDQFLGVPYAAPPLAESRFRAPEPLNWTGTWDAS 2170
Cow            SVPIATHGQLLG-RSQAIQVGTSWKPVDQFLGVPYAAPPLGEKRFRAPEHLNWTGSWEAT 2240
Panda          SVTIAPYGRLLG-RSQAVQVGSSWRQVDQFLGVPYAAPPLAESRFQAPEPLNWTGSWDAT 2240
Dog            SVTITPHGQLLG-RSQAIQVGTSWKQVDQFLGVPYATPPLAESRFRAPEPLNWTGSWDAT 2239
Horse          SVTIATHGQLLG-RSQAIRVGTSWKQVDQFLGVPYASPPLAESRFRAPEPLNWTGPWDAT 2239
Opossum        TVFIGTQGTLKG-KSEAIQVGAEWKRIIQFFGVPYAAPPLAQRRFSAPESFNWTGTWEAT 2243
Zebra finch    RVYIPSHGYLMG-KSQVIHVGSGWRNISQFLGIPYAAPPLGERRFCPPEPLAWLEAWNAT 2292
Sea urchin     ------------------------------------------------------------
Amphioxus      PRPTTSPSPRETICRLKKRRGPCRAKISRYYYLPSRDTCVKFRFGGCGGNANNFHSWAEC 1933
```

```
Zebrafish      FSRSSCLQPGD---LTDSTSSEDCLYLNVFVASSVEKNVPVLVFFHNS-------ASDLL 2141
Fugu           KPRPSCIQPGD---SETAASSEDCLYLNIFTPAQRRGHVPVLVFFTNLGAY---QSSQML 2048
Rat            KLRSSCWQPGT-RTPTPPQISEDCLYLNVFVPENLVSNASVLVFFHNTVEMEGSGGQLNI 2298
Mouse          KPRASCWQPGT-RTPTPPQINEDCLYLNVFVPENLVSNASVLVFFHNTMEMEGSGGQLTI 2296
Human          KPRASCWQPGT-RTSTSPGVSEDCLYLNVFIPQNVAPNASVLVFFHNTMDREESEGWPAI 2298
Marmoset       KLRASCWQPGT-RTSMSPGVSEDCLYLNVFIPQNVAPNTSVLVFFHNTMEGKGSEGWPAI 2250
Pig            KPRASCWQPGI-RPATAPGVSEDCLYLSVFVPQSLTPNSSVLVFFHNGAEG---PLAMAV 2226
Cow            KPRARCWQPGI-RTPTPPGVSEDCLYLNVFVPQNMAPNASVLVFFHNAAEGKGSGDRPAV 2299
Panda          KPRASCWQPGT-RAPASPGVSEDCLYLNLFVPQNVAPNASVLVFFHNAMEGKGAGGQLAI 2299
Dog            KPRASCWQPGT-RTLESPGVDEDCLYLNVFVPQNVAPNASVLVFFHNTLEGRGSEGPLAI 2298
Horse          KPRASCWQPGA-RTPLSPGLSEDCLHLNVFVPQNVAPGASVLVFFHNSMEGRVSEGQLAL 2298
Opossum        VPRAKCWQPGFGKTSSSSTVSEDCLYLNVFVPQNLGQNASVLVFFHNADENGRRRSQLDL 2303
Zebra finch    AARAACWQPGD-GEAPSQSVSEDCLYLHIFVPATTVKNMSVLVFFHNGGSYNAEEGKTTL 2351
Sea urchin     ------------------------------------------------------------
Amphioxus      METCTERGEAPKAPQPGPAVNPARETPRPEVPSYMGDSCILPAEPGPCQEAHSRWHFDPV 1993

Zebrafish      DGSYLAAVGNIIVVTASFRVAAFGFLSAGSSALPGNYGLQDQAAALGWVQKNIALFGGDP 2201
Fugu           DGSILAAVGNIVVVTASYRVAAFGFLSTESSGLHGNYGLSDQEAVLHWVNAHISLVGGDN 2108
Rat            DGSILAAVGNLIVVTANYRLGVFGFLSSGSDEVAGNWGLLDQVAALTWVQTHIGAFGGDP 2358
Mouse          DGSILAAVGNFIVVTANYRLGVFGFLSSGSDEVAGNWGLLDQVAALTWVQSHIGAFGGDP 2356
Human          DGSFLAAVGNLIVVTASYRVGVFGFLSSGSGEVSGNWGLLDQVAALTWVQTHIRGFGGDP 2358
Marmoset       DGSFLAAVGNLIVVTASYRVGVFGFLSSGSGEVSGNWGLLDQVAALTWVQTHIRAFGGDP 2310
Pig            DGSFLAAVGNLIVVTASYRTGVFGFLSSGSSEVSGNWGLLDQVAALTWVQTHIGVFGGDP 2286
Cow            DGSFLAAVGNLIVVTASYRTGIFGFLSSGSSELSGNWGLLDQVVALTWVQTHIQAFGGDP 2359
Panda          DGSFLAAIGNLIVVTAGYRVGVFGFLSSGSGEVSGNWGLLDQRAALTWVQTHIRVFGGDP 2359
Dog            DGSYLAAIGNLIVVTAGYRVGIFGFLSSGSGELSGNWGLLDQLAALTWVQTHIGVFGGDP 2358
Horse          DGSFLAAVGNLIVVTAGYRVGVFGFLSSGSSEVSGNWGLLDQVAALTWVHTHIGAFGGDP 2358
Opossum        EGSFLAAVGDIIVVTANYRVGIFGFLSTGSKEASGNWGLLDQVAALKWVQAHIGSFGGDP 2363
Zebra finch    DGSYLAAIGNVIVVTANYRVGVFGFLSTGSAEASGNAGLWDQLAALRWVQQNIASFGGDP 2411
Sea urchin     ------------------------------------------------------------
Amphioxus      N------------------RVCQEFQYGGCGGNTNRFGSWRECTIKCLGQQAVHCGGCQH 2035

Zebrafish      TKVTVGAERNGADIASLHLTSP--SASSLFSRALLMGGSVFSPAVVMSSSKAQAQTSSLA 2259
Fugu           ERVTVGAEQRGADITSLHLLS----SSPRFQRMLLMGGSVFSPSLLQTPPSSRREALQLA 2164
Rat            QRVTLAADRGGADVASIHLLITRPTRLQLFRKALLMGGSALSPAAIISPDRAQQQAAALA 2418
Mouse          QRVTLAADRSGADVASIHLLISRPTRLQLFRKALLMGGSALSPAAIISPERAQQQAAALA 2416
Human          RRVSLAADRGGADVASIHLLTARATNSQLFRRAVLMGGSALSPAAVISHERAQQQAIALA 2418
Marmoset       RRVSLAADRGGADVASIHLLTARATNSRLFRRAVLMGGSALSPAAIISHERAQQQAVALA 2370
Pig            RRVALAADRGGADVAGIHLLTSRATNSRLFRRAVLMGGSVLSPAAVIRPDRAQQQAAALA 2346
Cow            RRVTLAADRGGADIASIHLVTTRAANSRLFRRAVLMGGSALSPAAVIRPERARQQAAALA 2419
Panda          RRVTLAADRGGADVASIHLLTTRAADSRLFRRVMLMGGSAFSPAAVISQGRAQQQAAALA 2419
Dog            RRVTLAADRGGADVASIHLLTTRAADSRLFRRAILMGGSAFSPAVVISQGRAQEQAAALA 2418
Horse          RHVALAADRGGADVASIHLLTTRTANSRLFRRAVLMGGSALSPAAVISQERAQQQAAAFA 2418
Opossum        RKVSVAADRGGADIASIHLVTT-TANSRLFKRAILMGGSVLSPAATISQKRAQEQAAALA 2422
Zebra finch    GRVSLGAARGGADVASVHLLTD-MAGADLFSRLLLMGGSAFSPASIITTRRAQTQAAALA 2470
Sea urchin     ------------------------------------------------------------
Amphioxus      ERHGLHRGYCNNQFVMTGRVSKLELTPSWRVVTMVTSSEMISKRGGLRSIEYQGRDLAVQ 2095

Zebrafish      RELDCP-AADPSQLLDCLRSKPAHSINAAQTKLLAVSGPLQAWSPVVDGNVVREKPSVAL 2318
Fugu           TELGCFDNLNDMEMAACLRAIPAHELNIAQTKLLAVSGPFQSWSPVHQP----------T 2214
Rat            KEVGCP-NSSVQEVVSCFRQKPANILNEAQTKLLAVSGPFHYWGPVVDGQYLRELPSRRL 2477
Mouse          KEVGCP-TSSIQEVVSCLRQKPANILNDAQTKLLAVSGPFHYWGPVVDGQYLRELPSRRL 2475
Human          KEVSCP-MSSSQEVVSCLRQKPANVLNDAQTKLLAVSGPFHYWGPVIDGHFLREPPARAL 2477
Marmoset       KEVSCP-MSSSQEVVSCLRQKPASVLNDAQTKLLAVSGPFHYWSPVVDGQFLREAPARAL 2429
Pig            KEVGCP-PRPSQKWCPASAR-ACQPPHDAQSKLLAVSGPFHYWGPVVDGQLLREAPARAL 2404
Cow            KEVGCP-SSSVQEMVSCLRQEPARILNDAQTKLLAVSGPFHYWGPVVDGQYLRETPARVL 2478
Panda          EEISCP-TSSIQEMVSCLRQKPASVLNDAQTKLLAVSGPFHYWGPVVDGQYLREAPARAL 2478
Dog            EEIGCP-TSSTQELVSCLRQKPASILNDAQTKLLAVSGPFHYWGPVVDGQYLREAPARAL 2477
Horse          KEVGCP-TSSIQEMISCLRQEPANVLNDAQTKLLAVSGPFHYWGPVVDGQYLREAPARAL 2477
Opossum        GEVNCP-TSPEGDMMSCLRQKPADILNNAQTKLLAISGPFHYWGPVVDGVYLQETPTRTL 2481
Zebra finch    EDVGCP-SSTSEEIVACLRQLPARVLNDAQTKLLAISGPFQYWGPVMDGIYLREPLAKAL 2529
Sea urchin     ------------------------------------------------------------
Amphioxus      VLLPRN--------YNNKHGCPCYDVARGAEYVMMGRVDGNGYGMVDEKGYMAELNNKYA 2147
```

```
Zebrafish       QSGRFHKAEILLGSSFEDGLISRAKNIKN-FEQLQGRADSKTAFYAALSNSLGGDDANAF 2377
Fugu            NTSSFQRVDLLIGTSQHDSLITRARRLKD-LGDLLGHADGKTAFYEALSRSLGSVTGSEQ 2273
Rat             KRPLPVKVDLLIGGSQDDGLINRAKAVKQ-FEESQGRTNSKTAFYQALQNSLGGEDSDAR 2536
Mouse           KRPLPVKVDLLIGGSQDDGLINRAKAVKQ-FEESQGRTNSKTAFYQALQNSLGGEDSDAR 2534
Human           KRSLWVEVDLLIGSSQDDGLINRAKAVKQ-FEESRGRTSSKTAFYQALQNSLGGEDSDAR 2536
Marmoset        KRTLQVEVDLLIGSSQDDGLINRAKAVKQ-FEESQGRTSSKTAFYQALQNSLGGEDSDAR 2488
Pig             QRPPRAKLDLLIGSSQDDGLIDRAKAVKR-FEESQGRTSSKTAFYQALQNSLGGEAGDPG 2463
Cow             QRAPRVKVDLLIGSSQDDGLINRAKAVKQ-FEESQGRTSSKTAFYQALQNSLGGEAADAG 2537
Panda           QRTPRAKVDLLIGSSQDDGLINRAKAVKQ-FEESQGRTSSKTAFYQALQNSLGGEDADAG 2537
Dog             QRTLRAKVDLLIGSSQDDGLINRAKAVKQ-FEESQGRTSSKTAFYQALQNSLGGEDADAG 2536
Horse           QRFPRAKVDLLIGSSQDDGLINRAKAVKQ-FEESQGRTSSKTAFYQALQNSLGGEEADAG 2536
Opossum         QRSPRMKVDVLIGSAQRDGLINRAKAIKKSFQESQGRTSSKTAFYQALQNSLGGENSDAS 2541
Zebra finch     QRPRLRKVDLLIGSAQQDGLISRAKAIKK-FEESQGRANSKTAFYQALQNSLGGEESNSL 2588
Sea urchin      ------------------------------------------------------------
Amphioxus       RRLQQLKANPFSCS---YSYHDTSRQGSN-----------------PYARNTGHNRQNSR 2187


Zebrafish       VKEAATWFYSLQHSPTPSGYNVFSRALENATRDLFIICPTVDMAEFWAANTQTGVYMYHL 2437
Fugu            LKEVAVWFYSLDHSPTASGYNLFSRALDNATRDLFIICPSLRMVSHWAKHKAS-AFLYHQ 2332
Rat             ILAAAIWYYSLEHS--TDDYASFSRALENATRDYFIICPIVNMASLWARRTRGNVFMYHV 2594
Mouse           ILAAAVWYYSLEHS--TDDYASFSRALENATRDYFIICPMVNMASLWARRTRGNVFMYHV 2592
Human           VEAAATWYYSLEHS--TDDYASFSRALENATRDYFIICPIIDMASAWAKRARGNVFMYHA 2594
Marmoset        VEAAATWYYSLEHS--SDDYASFSRALENATRDYFITCPIIDMASAWAKRTRGNVFMYHA 2546
Pig             VQAAATWYYSLEHD--TDDYASFSRALEAATRDYFIICPVIDMASHWARTARGNVFMYHA 2521
Cow             VQAAATWYYSLEHD--SDDYASFSRALEQATRDYFIICPVIDMASHWARTVRGNVFMYHA 2595
Panda           VRAAATWYYSLEHS--TDDYASFSRALENATRDYFITCPVIDMASHWARRARGNVFMYHA 2595
Dog             VRAAATWYYSLEHS--TDDYASFSRALENATRDYFITCPVIDMASHWARRARGNVFMYHA 2594
Horse           VQAAATWYYSLEHS--ADDYASFSRALENATRDYFITCPVIDMASHWARRARGNVFMYHA 2594
Opossum         IEVAATWYYSLEHS--TEDYSSFSRALENATRDYFISCPIIDMASHWARATRGNVYMYYV 2599
Zebra finch     IEDAATWYYSLEHS--TDDYSSFSRALENATRDQFITCPIINMASYWAAASQGNVFMYHV 2646
Sea urchin      ------------------------------------------------------------
Amphioxus       TNPQNPRSYPQNPQ--PNPYPSSPRSYPQNPQAR-----------PQNPQPQNPQPYPQY 2234


Zebrafish       PENAAYNSVDLSVPMDVQYLFGVPLAAEKRDLFSYKEKTFTLQIMNYMANFIKSGNPNLP 2497
Fugu            PLSSAHSRAETSVPLDVQLMFGSPYHPINIQRFTRSDRRLSLATMTYFSTFVRTGNPNP- 2391
Rat             PESYGHG--SLELLADVQYAFGLPFYSAYQGYFSTEEQSLSLKVMQYFSNFIRSGNPNYP 2652
Mouse           PESYGHG--SLELLADVQYAFGLPFYSAYQGQFSTEEQSLSLKVMQYFSNFIRSGNPNYP 2650
Human           PENYGHG--SLELLADVQFALGLPFYPAYEGQFSLEEKSLSLKIMQYFSHFIRSGNPNYP 2652
Marmoset        PKSYGHG--SLELLVDVQFAFGLPFYPAYEGQFSLEEKSLSLKIMQYFSHFIRSGNPNYP 2604
Pig             PESYSHG--SLELLADVRYAFGLPFYPAYEGQFTQEEKSLSLKIMQYFSNFVRSGNPNYP 2579
Cow             PESYSHS--SLELLTDVLYAFGLPFYPAYEGQFTLEEKSLSLKIMQYFSNFIRSGNPNYP 2653
Panda           PESYGHS--SLDLLADVRSAFGLPFYPAYEGQFTLEEKSLSLKIMQFFSNFIRSGNPNYP 2653
Dog             PESYDHR--SLELLADVQYAFGLPFYPVYEGQFTLEEKSLSLKIMQFFSNFVRSGNPNYP 2652
Horse           PESHGHS--SLDLLADVQYAFGLPFHPAYEGQFTLEEKSLSLKIMQYFSNFVRSGNPNYP 2652
Opossum         PETYAQN--SLELLADVQYAFGLPFHPQYKQQFTLEEKNLSLKIMQYFSNFIKTGNPNYP 2657
Zebra finch     PENEGHY--AVEFLLDVQYAFGLPFYPKYEEQFTDEEKGLSLQIMQYISNFINSGNPNYL 2704
Sea urchin      ------------------------------------------------------------
Amphioxus       PRSSPQNPQPYPQNPQPYPQNPQPNPQDPRSNPQNPQPYPQNPYLYPQNPQPQNPQPYPQ 2294


Zebrafish       LAASRTSFGKFLPPWPQFMPHVGGRAYKELSSTLVNRKNLQRSQCSFWSQYVPALTSSTA 2557
Fugu            ---SHLWAESVLPQWPQVVSSDTPLTYLELSPTLKHNEGLSQRSCSFWNQYVPALTSSTA 2448
Rat             HEFSQKAA-EFATPWPDFVPGAGGESYKELSAQLPNRQGLKKADCSFWSKYIQTLKD-AD 2710
Mouse           HEFSRKAA-EFATPWPDFIPGAGGESYKELSAQLPNRQGLKQADCSFWSKYIQTLKD-AD 2708
Human           YEFSRKVP-TFATPWPDFVPRAGGENYKEFSELLPNRQGLKKADCSFWSKYISSLKTSAD 2711
Marmoset        YEFSRKVP-DFASPWPDFVPSAGGENYKEFSTQLPNRQGLKKADCSFWSKYISSLKASAD 2663
Pig             HEFSRKAP-EFAAPWPDFVPGDGAESYKELSVLLPNRQGLKKADCSFWSKYISSLKASAD 2638
Cow             HEFSRRAP-EFAAPWPDFVPRDGAESYKELSVLLPNRQGLKKADCSFWSKYIQSLKASAD 2712
Panda           HEFSRKAP-EFAAPWPDFVPRAGGESYKELSVLLPSRQGLKSADCSFWSKYIRSLKAAAA 2712
Dog             HEFSRKAS-ELAAPWPDFVPRTGGESYKEFSVLLPNRQGLKSADCSFWAKYIQSLKAVAD 2711
Horse           HEFSRKAP-EFAAPWPDFVPRAGRETYKEFSALLPNQQGLKKADCSFWSKYIQSLKALAG 2711
Opossum         YDFSRKIL-ETTALWPEFLPHTNGDNYKEFSPGLPNQRGLKKAECSFWSKYIQTLKASTG 2716
Zebra finch     HSFSRRML-GVMPAWPMYLSSDDGDNYKEFTVSLPTLKGLKKADCSFWSDYIRRLKASTG 2763
Sea urchin      ------------------------------------------------------------
Amphioxus       DPRSNPQNPQPYPQYPGYNPYNSGSNPQHPGSYPQDPRSHSQYPGHNPGPRHQAAPNTSA 2354
```

```
Zebrafish       KFSCGTSAEETGVQTPTQEPKPLTPASFTLTPSRQYN- 2572
Fugu            KFSLGTSAEETGVQTPTQSPKPLTLASFTLTPSRQY-P 2463
Rat             GAKDAQLTKSGEEDLEVGPGSEEDFSGSLEPVPKSYSK 2726
Mouse           GAKDAQLTKSEEEDLEVGPGLEEDLSGSLEPVPKSYSK 2724
Human           GAKGGQSAESEEEELTAGSGLREDLLSLQEPGSKTYSK 2727
Marmoset        GAKGGQLAESEEEELTAGSGVKEDLLSLQE---P-YSK 2675
Pig             EAEDEPLAESEEEDRPG---LAEDLLGLPELASKSYSK 2651
Cow             ETKDGPSADSEEEDQPAGSGLTEDLLGLPELASKTYSK 2728
Panda           GGEAGLSAESEEEDELADSGL------LGEPGSKSYSK 2722
Dog             EAKQELSAESEEDDEPADSGL------IGEPGSKSYSK 2721
Horse           EAKDELSAGSEEEDQPAGSGLREDLLGLPEPGSMSYSK 2727
Opossum         EAESQQPLVAEEE---LGSVELQSHLGVPEEGQKSYSK 2729
Zebra finch     SASGGEPGETPSEVTSLES--------QPLGDEVAYSR 2771
Sea urchin      --------------------------------------
Amphioxus       ARGRASYTHRSAHKR----------------------- 2369
```
